# Epstein-Barr virus nuclear antigen 3C (EBNA3C) interacts with the metabolism sensing C-terminal binding protein (CtBP) repressor to upregulate host genes

**DOI:** 10.1371/journal.ppat.1009419

**Published:** 2021-03-15

**Authors:** Makoto Ohashi, Mitchell Hayes, Kyle McChesney, Eric Johannsen

**Affiliations:** 1 Department of Medicine, Division of Infectious Diseases, University of Wisconsin-Madison, Madison, Wisconsin, United States of America; 2 Department of Oncology, McArdle Laboratory for Cancer Research, School of Medicine and Public Health, University of Wisconsin-Madison, Madison, Wisconsin, United States of America; University of North Carolina at Chapel Hill, UNITED STATES

## Abstract

Epstein-Barr virus (EBV) infection is associated with the development of specific types of lymphoma and some epithelial cancers. EBV infection of resting B-lymphocytes *in vitro* drives them to proliferate as lymphoblastoid cell lines (LCLs) and serves as a model for studying EBV lymphomagenesis. EBV nuclear antigen 3C (EBNA3C) is one of the genes required for LCL growth and previous work has suggested that suppression of the CDKN2A encoded tumor suppressor p16^INK4A^ and possibly p14^ARF^ is central to EBNA3C’s role in this growth transformation. To directly assess whether loss of p16 and/or p14 was sufficient to explain EBNA3C growth effects, we used CRISPR/Cas9 to disrupt specific CDKN2A exons in EBV transformed LCLs. Disruption of p16 specific exon 1α and the p16/p14 shared exon 2 were each sufficient to restore growth in the absence of EBNA3C. Using EBNA3C conditional LCLs knocked out for either exon 1α or 2, we identified EBNA3C induced and repressed genes. By trans-complementing with EBNA3C mutants, we determined specific genes that require EBNA3C interaction with RBPJ or CtBP for their regulation. Unexpectedly, interaction with the CtBP repressor was required not only for repression, but also for EBNA3C induction of many host genes. Contrary to previously proposed models, we found that EBNA3C does not recruit CtBP to the promoters of these genes. Instead, our results suggest that CtBP is bound to these promoters in the absence of EBNA3C and that EBNA3C interaction with CtBP interferes with the repressive function of CtBP, leading to EBNA3C mediated upregulation.

## Author summary

Epstein-Barr virus (EBV) is a gammaherpesvirus that establishes lifelong infection in about 95% of adult humans. EBV infection is usually benign, but can rarely result in several different malignancies, particularly lymphomas. EBV infection of resting B-lymphocytes in the laboratory drives them to proliferate as lymphoblastoid cell lines (LCLs), a model for EBV lymphomagenesis. In this manuscript we study how one EBV protein expressed in LCLs, EBNA3C, contributes to B lymphocyte transformation. Prior work has established that EBNA3C turns off the CDKN2A gene, but there is disagreement regarding the relative importance of silencing the two CDKN2A gene products: p14 and p16. Using a CRISPR/Cas9 gene editing strategy we confirm that p16 knock-out rescues LCL growth in the absence of EBNA3C even in the presence of wildtype p14. We then use these knock-out LCLs to identify EBNA3C regulated genes and uncover extensive growth-independent changes in B lymphocytes due to the EBNA3C transcription factor. We also discover an unexpected role for the CtBP repressor protein in EBNA3C gene upregulation. Contrary to prior models, we do not observe CtBP recruitment to target genes by EBNA3C. Instead, our data are consistent with EBNA3C interfering with the ability of pre-bound CtBP to repress genes.

## Introduction

Epstein-Barr Virus (EBV) is a herpesvirus that has established lifelong infection in approximately 90% of the human population [1]. Primary EBV infection is the cause of heterophile positive infectious mononucleosis, a self-limited disease characterized by fever, pharyngitis, lymphadenopathy, and the appearance of atypical lymphocytes in the peripheral blood [2]. These lymphocytes are predominantly CD8+ T cells reactive against EBV infected B lymphocytes and are essential for recovery from primary infection [3]. Thereafter, EBV establishes chronic latent infection in memory B cells and is periodically shed in oral secretions [4]. In a minority of individuals and especially in the immunosuppressed, latent EBV infection results in lymphomas, including Hodgkin, Burkitt and Diffuse large B-cell lymphoma, primary CNS lymphoma, and post-transplant lymphoproliferative disease. EBV is also associated with epithelial malignancies, including nasopharyngeal carcinoma and about 10% of gastric cancers [5].

The ability of latent EBV infection to convert resting B lymphocytes *in vitro* into growth transformed lymphoblastoid cell lines (LCLs) has proven a critical model for understanding how EBV causes lymphoma, particularly in the immunosuppressed [6,7]. LCLs are characterized by expression of latency associated viral proteins, including 6 nuclear proteins (EBNA1, EBNA2, EBNA3A, EBNA3B, EBNA3C, and EBNALP), 3 membrane proteins (LMP1, LMP2A, and LMP2B), non-coding RNAs (EBERs and BARTs) and at least 30 miRNAs [1]. Extensive study of LCLs has established that these latent gene products activate growth and survival signals characteristic of antigen stimulated B lymphoblasts. Reverse genetic analysis has demonstrated that some latent genes are essential for LCL transformation, others contribute to transformation efficiency, whereas some are completely dispensable [8].

An essential role for the EBNA3C nuclear protein was first demonstrated when truncation of the EBNA3C ORF via a stop codon at position 366 resulted in a non-transforming virus [9]. EBNA3C lacks a DNA-binding domain, but targets host chromatin through interactions with host transcription factors, especially RBPJ, a component of the Notch signaling pathway. ChIP-seq studies unexpectedly demonstrated that most EBNA3C bound sites lack RBPJ co-binding, implying that other host transcription factors are targeted by EBNA3C [10,11]. The full extent of these transcription factors is unknown, but we have demonstrated the importance of IRF4 in this role [10]. Additionally, EBNA3C interacts with a number of transcriptional repressors and activators which are proposed to regulate transcription of host genes when recruited to EBNA3C bound sites [12–15]. Notable among these are the C terminal binding proteins (CtBP1 and CtBP2), metabolic sensing transcriptional repressors whose dimerization and repressive activities are dependent upon NADH binding [16]. EBNA3C has been shown to interact with CtBP1 (the predominant form present in lymphocytes, hereafter referred to as CtBP) via a PLDLS motif in its C-terminus and this interaction appears to be important for some EBNA3C repressive effects, but dispensable for others [17].

Key insights implicating these EBNA3C transcriptional effects as being essential for its oncogenic activity were obtained using an EBV genome rendered conditional for EBNA3C activity by means of an in-frame fusion with a 4-hydroxytamoxifen specific-estrogen hormone receptor binding domain (EBNA3C-HT). EBNA3C-HT LCLs clearly demonstrated that EBNA3C activity was required to maintain LCL growth transformation phenotype [18]. Additionally, EBNA3C-HT LCLs could be trans-complemented with EBNA3C mutants to assess their ability to support growth [19,20]. This revealed that EBNA3C interaction with RBPJ was essential for growth and that an EBNA3C mutant defective for interaction with CtBP was impaired for growth maintenance [19,20]. EBNA3C-HT LCLs provided an additional insight into EBNA3C’s role in transformation: growth arrest induced by EBNA3C inactivation was accompanied by upregulation of the CDKN2A tumor suppressor gene products p16^INK4A^ and p14^ARF^ and shRNA targeting of both p16 and p14 could restore LCL growth in the absence of EBNA3C [18,20]. In the presence of EBNA3C, the CDKN2A locus becomes epigenetically repressed via the deposition of the facultative heterochromatin mark H3K27me3 [21]. The details of how EBNA3C recruits the PRC2 complex to deposit this mark at the CDKN2A locus are incompletely understood. However, study of other EBNA3C repressed genes suggests PRC1 is first recruited to EBNA3C bound sites and, subsequently, PRC2 is recruited to the transcriptional start site of the target gene [22]. Additionally, co-expression of the EBNA3A nuclear protein and EBNA3C’s interactions with RBPJ and CtBP appear to be essential for CDKN2A repression [21,23].

EBNA3C regulation of other cell genes is likely important in EBV biology and disease. For example, EBNA3C collaborates with EBNA3A to repress BCL2L11 which may play a role in the pathogenesis of Burkitt lymphoma and contribute to transformation in other contexts by limiting apoptotic signaling [24]. EBNA3C is also thought to suppress plasma cell differentiation by repressing key mediators of this process including CDKN2C (p18^INK4C^) and PRDM1 (BLIMP) [25]. EBNA3C is also essential to overcome cellular DNA damage checkpoints, a major bottleneck during initial transformation of B lymphocytes into LCLs [26]. In addition, a large number of non-transcriptional EBNA3C effects have been reported, including promotion of Rb degradation by SCF ubiquitin ligases [27], *c-myc* stabilization [28], and inactivation of cyclinA-CDK complexes [29]. The extent to which these non-transcriptional mechanisms are important for LCL transformation and growth maintenance remains to be established.

The most direct evidence reported to date identifying CDKN2A repression as a key mechanism of EBNA3C mediated transformation is a study by Skalska et al [30]. They reported that an EBV genome deleted for EBNA3C could still transform B lymphocytes from an individual homozygous for p16-Leiden, a 19-base pair germ-line deletion in the CDKN2A second exon [30]. While this study strongly suggested that p16 was the essential target of EBNA3C, it was not possible to unequivocally establish that the CDKN2A mutation was responsible for the phenotype. Because only a single affected individual was tested, the possibility remained that this individual harbored additional uncharacterized mutations that allowed for LCL growth in the absence of EBNA3C. Additionally, the p16-Leiden mutation creates a p16-p14 fusion protein that was argued to be intact for all known p14 activities. However, a number of non-canonical p14 activities have subsequently been described which require sequences within the second CDKN2A exon. The extent to which this fusion protein is compromised for non-canonical p14 activities that may compliment loss of EBNA3C activity is unknown [31–33].

Regulation of genes other than CDKN2A by EBNA3C may also play critical roles, especially *in vivo*. Despite multiple reports [30,34,35], there has been little consensus regarding the identity of these EBNA3C regulated gene sets. This likely reflects, at least in part, context dependence of EBNA3C’s regulation of host genes. For example, EBNA3C is known to bind IRF4 and we have previously demonstrated that IRF4 is essential for EBNA3C binding to a subset of its regulatory sites [10,36]. In Burkitt lymphoma (BL) cells, which lack IRF4 expression, EBNA3C may regulate different target genes than in LCLs. Thus, expression of EBNA3C by infection or transfection of EBV negative BL cells may fail to capture many genes regulated by EBNA3C in LCLs. However, even the two prior studies that used LCLs had limited concordance: reporting 550 and 429 EBNA3C regulated genes with only 45 of these (~10%) in common [30,34]. An important difference between these two studies that may account for the discrepancy is that one utilized wildtype p16 LCLs whereas the other employed LCLs homozygous for p16-Leiden. To directly address these questions, we designed sgRNAs targeting CDKN2A exons and used them to knockout p16 shared or unique exons from EBNA3C-HT LCLs. This allowed us to directly demonstrate the requirement for p16 inhibition in EBNA3C mediated growth effects. Further, by trans-complementing these cell lines with EBNA3C mutants defective for RBPJ or CtBP binding we were able to assess the role of these cell proteins in regulating host genes other than the CDKN2A gene products. Our results revealed that the RBPJ interaction is more important than CtBP for most regulated genes, but also identified specific genes for which either the RBPJ or CtBP interaction was dispensable. Surprisingly, we found that interaction with the CtBP repressor was more important for EBNA3C’s ability to induce host genes than for EBNA3C mediated repression. We also could not find evidence that EBNA3C recruits CtBP to promoters as previously proposed. Instead, our results are consistent with a model in which EBNA3C binds to regulatory elements already occupied by CtBP and upregulates genes by interfering with CtBP mediated repression.

## Results

### Establishment and characterization of CDKN2A-exon2 knockout EBNA3C-HT LCLs by CRISPR/Cas9 gene editing

**Fig 1:**
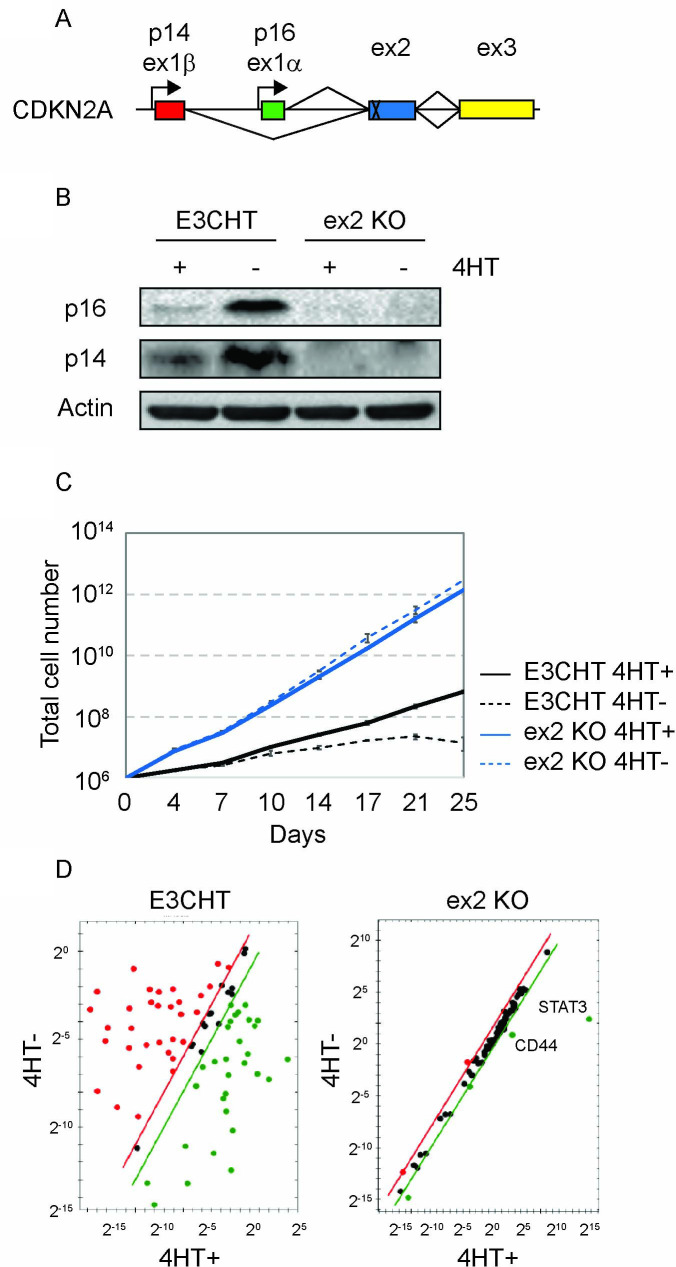
Establishment and characterization of CDKN2A-exon2 knockout EBNA3C-HT LCLs. (A) Schematic of the CDKN2A locus illustrating CRISPR/Cas9 targeting strategy within the context of p14 unique (1β), p16 unique (1α) and shared (2 and 3) exons. (B) Western blots of cell lysates from EBNA3C-HT LCLs (E3CHT) and CDKN2A-ex2-KO EBNA3C-HT LCLs (ex2-KO) cultured in the presence (+) or absence (-) of 4HT for 3 weeks and probed for p16, p14, and actin expression as indicated. (C) Growth curves for EBNA3C-HT LCLs (black lines) and CDKN2A- ex2-KO EBNA3C-HT LCLs (red lines) grown in the presence (solid lines) or absence (dashed lines) of 4HT. Cells were counted every 3 to 4 days, and diluted in fresh media to maintain a concentration of 200,000 cells/mL. The growth curves shown are from an experiment representative of two independent experiments. (D) TaqMan Array Human Notch Signaling results demonstrating increased specificity for EBNA3C regulated genes resulting from continued log phase growth in and CDKN2A-ex2-KO EBNA3C-HT LCLs (ex2-KO) compared to EBNA3C-HT LCLs (E3CHT) upon 4HT withdrawal. For each gene, the expression level relative to TBP control is plotted in the 4HT+ and 4HT- condition. Genes to the right of the diagonal lines are significantly upregulated, whereas those to the left are significantly downregulated in this assay. Genes experiencing the greatest fold change in the CDKN2A-ex2-KO EBNA3C-HT LCLs are indicated.

In order to directly assess the importance of p16 inactivation for EBNA3C’s role in supporting LCL growth, we designed an sgRNA targeting CDKN2A-exon2, the exon affected by the p16-Leiden deletion (Fig 1A). Using this sgRNA, we established CDKN2A knockout EBNA3C-HT LCLs via CRISPR/Cas9 co-expression from an oriP-based plasmid. Hygromycin resistant cells were selected and screened initially by PCR (S1A Fig). Sanger sequencing of ex2-KO-clone 3 revealed disruption of both exon2 alleles: one allele having a frame shift mutation due to insertion of a single C residue within the sgRNA target while the second had a 234bp deletion within the targeted region (S1B Fig). Resultant knockout cells no longer express Cas9-Flag in the absence of hygromycin selection (S1C Fig). This approach eliminates potential disadvantageous effects of ongoing Cas9 expression (e.g., DNA damage) in CDKN2A knockout EBNA3C-HT LCLs. The ex2-KO EBNA3C-HT LCLs did not express detectable p16 or p14 (Fig 1B) or experience growth arrest (Fig 1C) upon 4-hydroxytamoxifen (4HT) withdrawal. In contrast, these CDKN2A gene products were strongly detected upon 4HT withdrawal in the parental EBNA3C-HT LCL which ceased proliferating after about 2 weeks in the absence of 4HT. Notably, the ex2-KO EBNA3C-HT LCLs grew faster without 4HT than the parental line did in the 4HT+ condition. This growth impairment and the incomplete p14/p16 suppression is consistent with prior observations that EBNA3C-HT is hypomorphic relative to wildytpe EBNA3C [23,34].

As a preliminary test of whether the ex2-KO EBNA3C-HT system would identify EBNA3C regulated genes with greater specificity than the parental cell line, we assayed a panel of host genes using the Human Notch Signaling TaqMan Array. Although the majority of genes were significantly changed in the EBNA3C-HT LCL upon 4HT withdrawal, only two highly expressed genes scored as EBNA3C regulated in the ex2-KO EBNA3C-HT LCLs: CD44 and STAT3 (Fig 1D). Although we could only confirm the former by RNA-seq (discussed in subsequent sections), it was evident that the absence of growth arrest in the CDKN2A ex2-KO EBNA3C-HT LCLs would permit detection of EBNA3C regulated genes with much increased specificity.

### Construction of a p16 specific knockout in EBNA3C-HT LCLs

**Fig 2:**
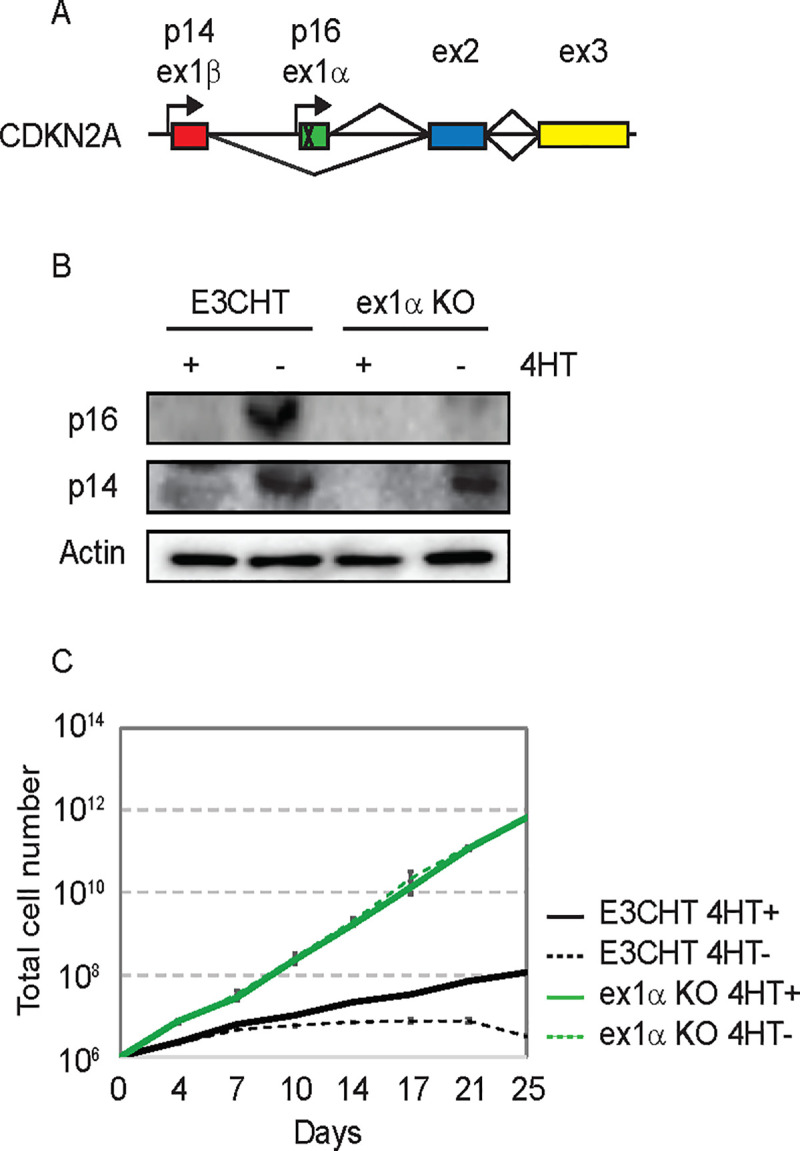
Establishment and characterization of CDKN2A-exon1α knockout EBNA3C-HT LCLs. (A) Schematic of the CDKN2A locus illustrating CRISPR/Cas9 strategy specifically targeting the p16 unique exon 1α. (B) Western blots of cell lysates from EBNA3C-HT LCLs (E3CHT) and CDKN2A-ex1α-KO EBNA3C-HT LCLs (ex1α KO) cultured in the presence (+) or absence (-) of 4HT for 3 weeks and probed for p16, p14, and actin expression as indicated. (C) Growth curves for EBNA3C-HT LCLs (black lines) and CDKN2A-ex1α-KO EBNA3C-HT LCLs (blue lines) grown in the presence (solid lines) or absence (dashed lines) of 4HT. Cells were counted every 3 to 4 days, and diluted in fresh media to maintain a concentration of 200,000 cells/mL. The growth curves shown are from an experiment representative of two independent experiments.

The p16 Leiden mutation results in a p14-p16 fusion protein that retains canonical p14 activity in the p53 pathway. The ability of EBNA3C deleted EBV to transform B lymphocytes from an individual homozygous for p16 Leiden led to the assertion that p14 repression is not an essential EBNA3C function. Although we observed loss of p14 signal in Fig 1B, our ex2-KO EBNA3C-HT LCLs also have the potential to express p14 fusion proteins that lack the C-terminal epitope recognized by our p14 antibody (S2A Fig). Consistent with prior reports, we could not detect any p14 induced effect on p53 protein levels in the ex2-KO EBNA3C-HT LCLs or the parental EBNA3C-HT LCLs (S2B Fig). However, non-canonical p14 functions have been mapped to CDKN2A exon2, that may be important for complementing loss of EBNA3C activity [31–33]. In order to directly assess whether p16 loss alone was sufficient to allow EBNA3C independent growth, we constructed an sgRNA targeting the p16 unique exon1α (Fig 2A). Using the same approach as for exon 2, we screened candidate clones and sequencing revealed a 24bp deletion of exon1α that included the initiator methionine codon (S3 Fig). Western blotting confirmed loss of p16 expression in the ex1α-KO EBNA3C-HT LCLs, but strong induction of the p14 gene product upon 4HT withdrawal (Fig 2B). Importantly, the ex1α-KO EBNA3C-HT LCLs continued to proliferate rapidly in the absence of 4HT. Again, we observed that the knockout cell line grew more rapidly in the absence of 4HT than the parental clone in the presence of 4HT (Fig 2C). These results are consistent with EBNA3C suppression of p16 being sufficient to explain its role in LCL growth maintenance.

### Identification of EBNA3C regulated genes using p16-KO EBNA3C-HT LCLs

**Fig 3:**
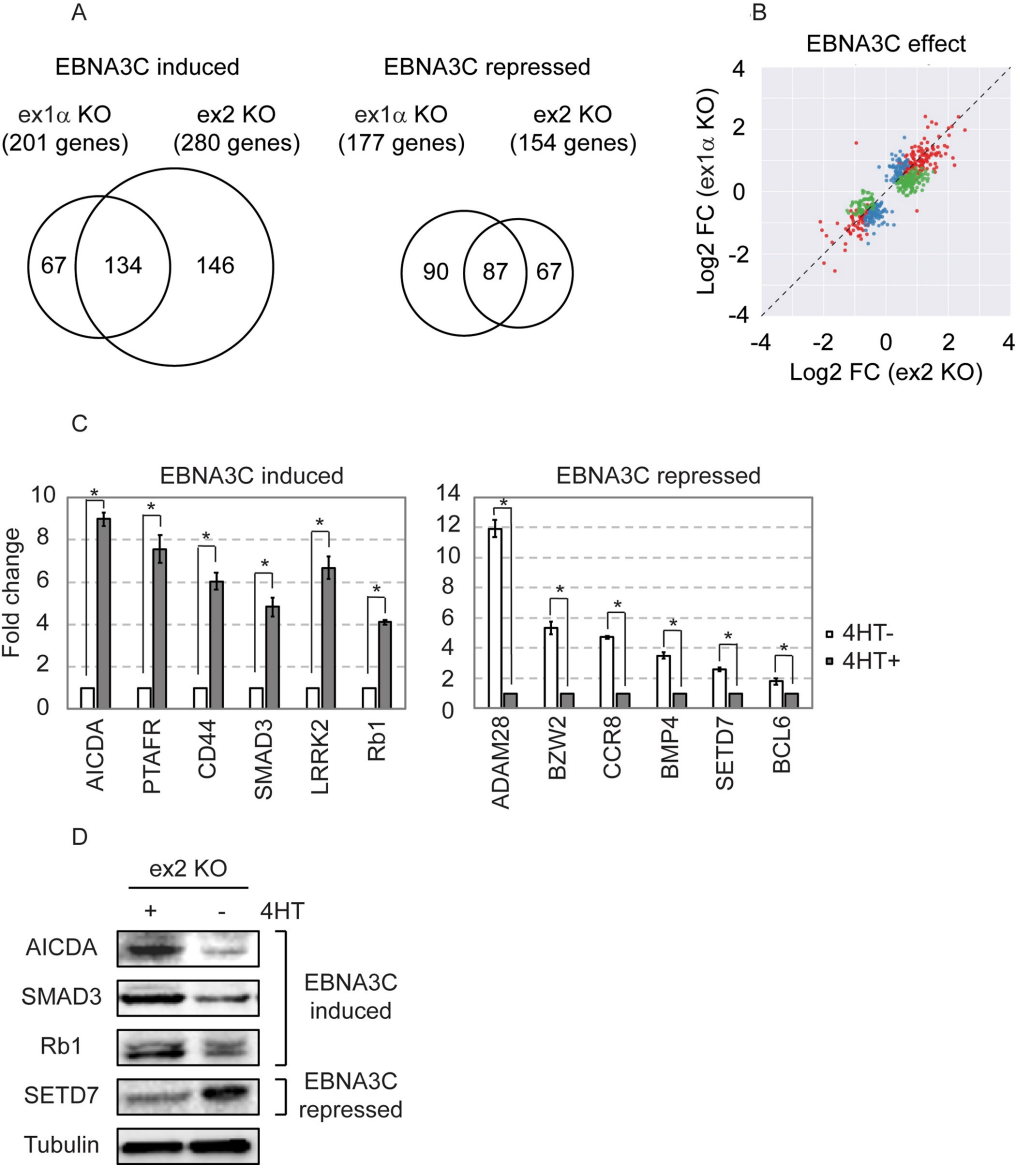
Identification of EBNA3C regulated genes by RNA-seq in p16-KO EBNA3C-HT LCLs. (A) Venn diagrams summarizing results of differential expression analysis for EBNA3C regulated genes in the CDKN2A-ex1α-KO and CDKN2A-ex2-KO EBNA3C-HT LCLs. This identified 134 genes that were significantly EBNA3C induced in both cell lines and 87 genes significantly repressed. (B) Scatter plot comparing extent of regulation of each gene by EBNA3C in the ex1α-KO vs. ex2-KO EBNA3C-HT LCLs. Color indicates whether the gene was found to be EBNA3C regulated in the ex1α-KO cell line (blue) in the ex2-KO cell line (green) or both (red); FC = fold change. (C) Confirmation qRT-PCR assays measuring fold change for indicated EBNA3C regulated genes in ex2-KO EBNA3C-HT LCLs grown in the presence (4HT-) or absence (4HT+) of EBNA3C activity. Fold change was calculated with delta-delta-Cq methods as described in materials and methods. The experiment shown is representative of two independent biological replicates and * indicates statistically signficifant differences (p-value < 0.05 by two-sample t-test). (D) Western blots probed for indicated EBNA3C regulated genes of cell lysates from ex2-KO EBNA3C-HT LCLs grown in the presence (+) or absence (-) of 4HT.

In an effort to better define the cellular targets of EBNA3C, we assessed differential gene expression in our ex1α- and ex2-KO EBNA3C-HT LCLs (collectively referred to as p16-KO EBNA3C-HT LCLs). For these experiments, 4 replicates of each cell line were cultured in the presence 4HT or for 2 weeks after 4HT withdrawal prior to harvesting for RNA-seq. Reads were aligned to the human genome (GRCh38) as described in the materials and methods and differential gene expression determined with DESeq2. Using a false-discovery rate of 5% (q < 0.05), we identified 201 and 280 EBNA3C induced genes in each cell line with 134 of these genes being identified in both cell lines (Fig 3A and S1 and S2 Tables). This approach also identified 177 and 154 EBNA3C repressed genes with an overlap of 87 genes (Fig 3A). Importantly, there was a high degree of concordance between the extent of EBNA3C upregulation or downregulation in both cell lines. In nearly every case, when a gene met statistical criteria in only one cell line, the effect of EBNA3C on the same gene in the other cell line was similar, but to a slightly lesser degree (Fig 3B). Only two genes were exceptional: Diacylglycerol kinase iota (DGKI) was upregulated in ex1α-KO cells, but downregulated in ex2-KO cells, whereas Limbic System Associated Membrane Protein (LSAMP) was downregulated in ex1α-KO cells, but upregulated in ex2-KO cells. These potentially interesting exceptions may be indirectly regulated via p14 effects. Nevertheless, it is likely that most of the genes identified as regulated in either cell line are *bona fide* EBNA3C target genes and that the intersections in the Venn diagrams represent the most robustly EBNA3C regulated genes.

For select EBNA3C regulated genes, we performed confirmatory qRT-PCR and immunoblotting. Six of six upregulated genes, including CD44 which was also found to be EBNA3C upregulated by TaqMan Array (Fig 3C, left panel), were confirmed by qRT-PCR. Six downregulated genes were also confirmed by this approach (Fig 3C, right panel). For four genes, we obtained reliable western blotting antibodies and, in each case, observed corresponding changes at the protein level as well (Fig 3D). We also compared our putative EBNA3C regulated genes with previously published data (S3 Table). There was good agreement between our study and that of Skalska et al. [30], who reported 34 of our 134 (25%) EBNA3C induced genes and 25 of our 87 (29%) EBNA3C repressed genes using p16 null LCLs. By contrast, a study by Zhao et al. [34], on which one of us is a co-author, used p16 intact LCLs and was less concordant with our findings, reporting 14 of our 134 (10%) EBNA3C induced genes and 13 of our 87 (15%) EBNA3C repressed genes. Moreover, that study identified 7 of our 134 EBNA3C upregulated genes as downregulated and 9 of our 87 downregulated genes as upregulated. There were no instances where the direction of EBNA3C gene regulation was discordant between our results and the Skalska study. This agreement is all the more remarkable considering they measured gene expression with Affymetrix arrays whereas our study employed RNA-seq. These comparisons and our results (Fig 1D) illustrate that p16 mediated growth arrest is a major confounder of attempts to identify direct EBNA3C target genes.

### Gene set enrichment analysis of EBNA3C regulated genes

**Fig 4:**
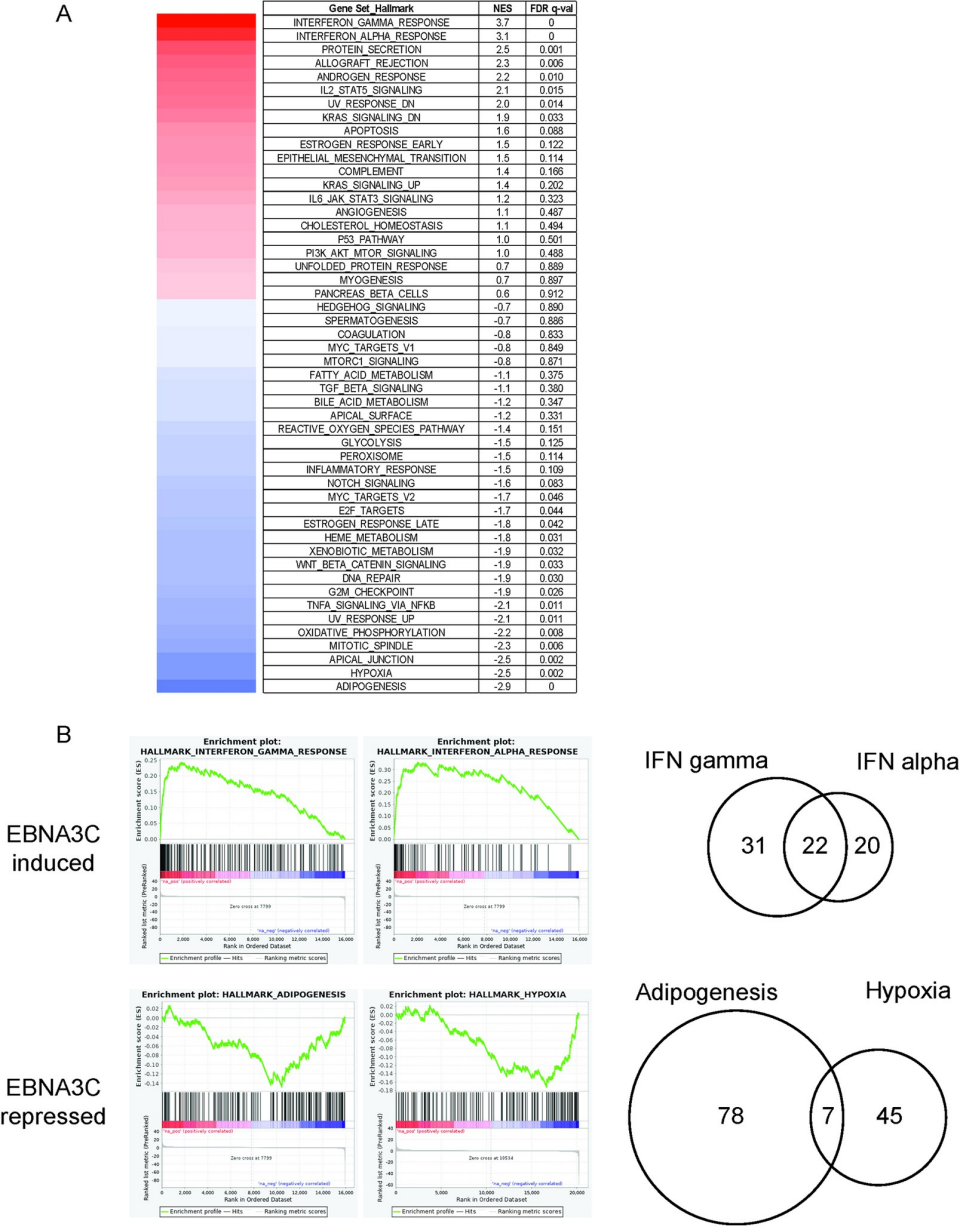
GSEA pathway analysis of EBNA3C regulated genes. (A) Normalized enrichment scores and false discovery rate (FDR) for the fifty Hallmark genes sets of MSigDB using pre-ranked EBNA3C regulated genes from ex2-KO EBNA3C-HT LCLs in the HT+ vs. HT- condition. (B) Running enrichment plots for the two sets with the highest positive (EBNA3C induced) and highest negative (EBNA3C repressed) enrichment scores. Venn diagrams at the right represent a comparison of the number of core enriched genes shared for the indicated gene sets.

In an effort to identify pathways targeted by EBNA3C for regulation, we compared our EBNA3C regulated genes against the Hallmark gene sets of MSigDB. This was done using GSEA and a pre-ranked EBNA3C regulated gene list based on the ex2-KO EBNA3C-HT LCL RNA-seq data (Fig 4A). The strongest positive enrichment scores were seen with the INTERFERON GAMMA RESPONSE (NES 3.7, q-val 0) and INTERFERON ALPHA RESPONSE (NES 3.1, q-val 0). Examination of the core enriched genes from each of these two signatures revealed that 22 of 53 genes responsible for the interferon gamma enrichment signal were among the 42 core enriched genes in the interferon alpha signal. Thus, these two “hits” likely represent the same biologic phenomenon: upregulation of interferon responsive genes by EBNA3C. We observed the strongest negative correlations with ADIPOGENESIS (NES -2.9, q-val 0) and HYPOXIA (NES -2.5, q-val 0.002). In contrast to the interferon signatures, the core enriched genes for these pathways exhibited minimal overlap (Fig 4B), suggesting that these are independent gene set correlations.

### Dependency of EBNA3C’s interaction with RBPJ and CtBP for cell gene regulation

**Fig 5:**
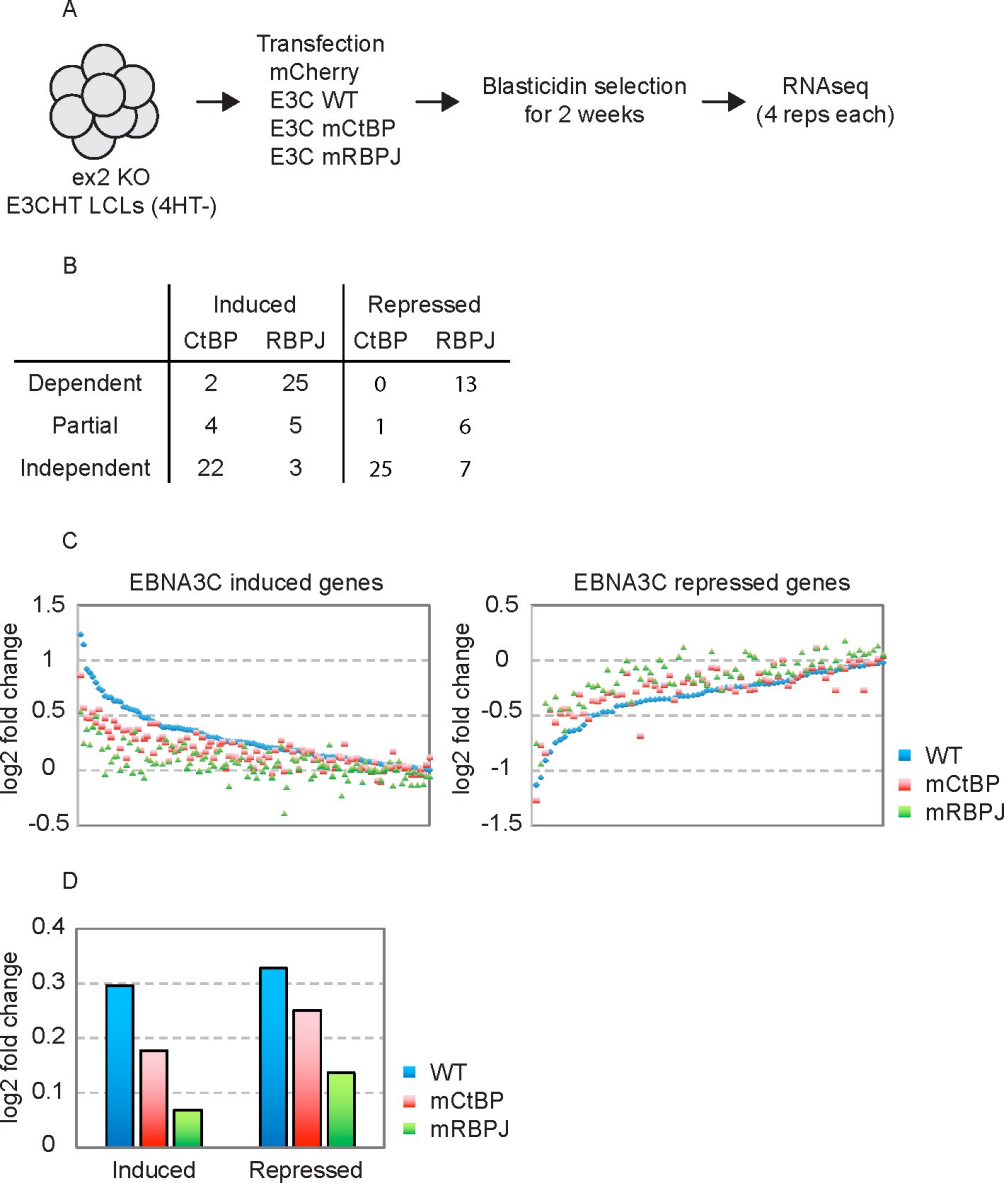
Dependency of EBNA3C interaction with RBPJ and CtBP for regulation of specific cell genes. (A) Schematic of experimental design to determine which EBNA3C regulated genes require EBNA3C interaction with RBPJ and/or CtBP. (B) Summary of extent of dependence upon RBPJ and CtBP interaction for EBNA3C repression or induction of specific host genes based on RNA-seq results from CDKN2A-ex2-KO EBNA3C-HT LCLs trans-complemented with specific EBNA3C mutants. For RBPJ, genes differentially expressed in E3CmRBPJ compared to E3C-WT were defined as dependent, genes differentially expressed in E3C-mRBPJ compared to control were defined as independent. Genes differentially expressed in E3C-mRBPJ compared to both E3C-WT and control were defined as partial. For CtBP, gene dependences were defined in the same manner using E3C-mCtBP compared to E3C-WT and control. (C) Log-2-fold change of EBNA3C repressed and induced genes is indicated for ex2-KO EBNA3C-HT LCLs trans-complemented with E3C-WT (blue), E3C-mCtBP (red), and E3C-mRBPJ (green). Only genes identified as high confidence EBNA3C targets and confirmed to be regulated by E3C-WT in this assay are shown. (D) Average log-2-fold change of EBNA3C repressed and induced genes from (C) in response to E3C-WT, E3C-mCtBP, and E3C-mRBPJ.

Previous studies have shown that EBNA3C must interact with RBPJ to regulate many target genes including CDKN2A, COBLL1 and AICDA [21–23,37] as well as the viral EBNA promoter (Cp) [38]. The CtBP interaction is dispensable for repression of the EBNA promoter [17] but is required for repression of CDKN2A [23]. However, the requirement for EBNA3C interaction with RBPJ and/or CtBP to regulate most host genes is unknown. In an effort to determine which EBNA3C target genes are regulated through these interactions, we performed trans-complementation assays with EBNA3C mutants defective for interaction with either RBPJ or CtBP. For these experiments, the ex2-KO EBNA3C-HT LCLs were cultured for 2 weeks in the absence of 4HT, and trans-complemented with EBNA3C wild type (WT), EBNA3C RBPJ binding mutant (mRBPJ), EBNA3C CtBP binding mutant (mCtBP), or mCherry protein (Cntl). As EBNA3C is not required for growth of ex2-KO LCLs, trans-complementation was maintained by blasticidin selection for 2 weeks prior to harvest and RNA-seq analysis (Fig 5A).

As performed previously, reads were mapped to GRCh38 using STAR and differentially expressed genes identified using DESeq2. We restricted this analysis to classification of the 134 EBNA3C induced and 87 EBNA3C repressed genes in Fig 3A. This identified 38 of these genes as RBPJ-dependent based on their differential expression between E3CmRBPJ and E3C-WT trans-complemented cells (Fig 5B and S4 Table). A total of 10 RBPJ-independent genes were defined as those differentially expressed between E3CmRBPJ and mCherry trans-complemented cells. We also identified 11 genes with intermediate phenotype (i.e., differentially expressed by E3CmRBPJ relative to both E3C-WT and mCherry) which we designated as partially RBPJ-dependent. Using a similar approach, we evaluated CtBP dependency. This identified 2 genes as CtBP-dependent, 5 as partially dependent, and 47 as CtBP-independent. These conservative criteria were employed because the additional variance observed in these RNA-seq data (presumably introduced by the need for blasticidin selection), prevented unambiguous classification of many EBNA3C regulated genes. We further examined the CtBP dependency of several EBNA3C regulated genes by qRT-PCR. For the CtBP-independent genes HHEX and IL16, we observed E3CmCtBP induction comparable to E3C-WT. In contrast, E3CmCtBP was clearly impaired for activation of the CtBP-dependent SMAD3 gene as well the partially dependent PARP9 gene (S4 Fig, left panel). We additionally confirmed the CtBP-independent mechanism of the EBNA3C repressed genes ANKMY2, GIMPA5, and GIMAP6 (S4 Fig, right panel).

To assess more broadly the effect of the RBPJ and CtBP binding mutants, we plotted the fold change relative to E3C-WT of each mutant for EBNA3C induced and repressed genes (Fig 5C). Two trends emerged from these data. First, E3CmRBPJ is more impaired for host gene regulation than E3CmCtBP. Second, the inability of E3CmCtBP to upregulate host genes to the same degree as E3C-WT (Fig 5D) suggested an unexpectedly important role for CtBP in EBNA3C mediated gene activation.

### The CtBP repressor is not recruited to regulatory elements by EBNA3C

**Fig 6:**
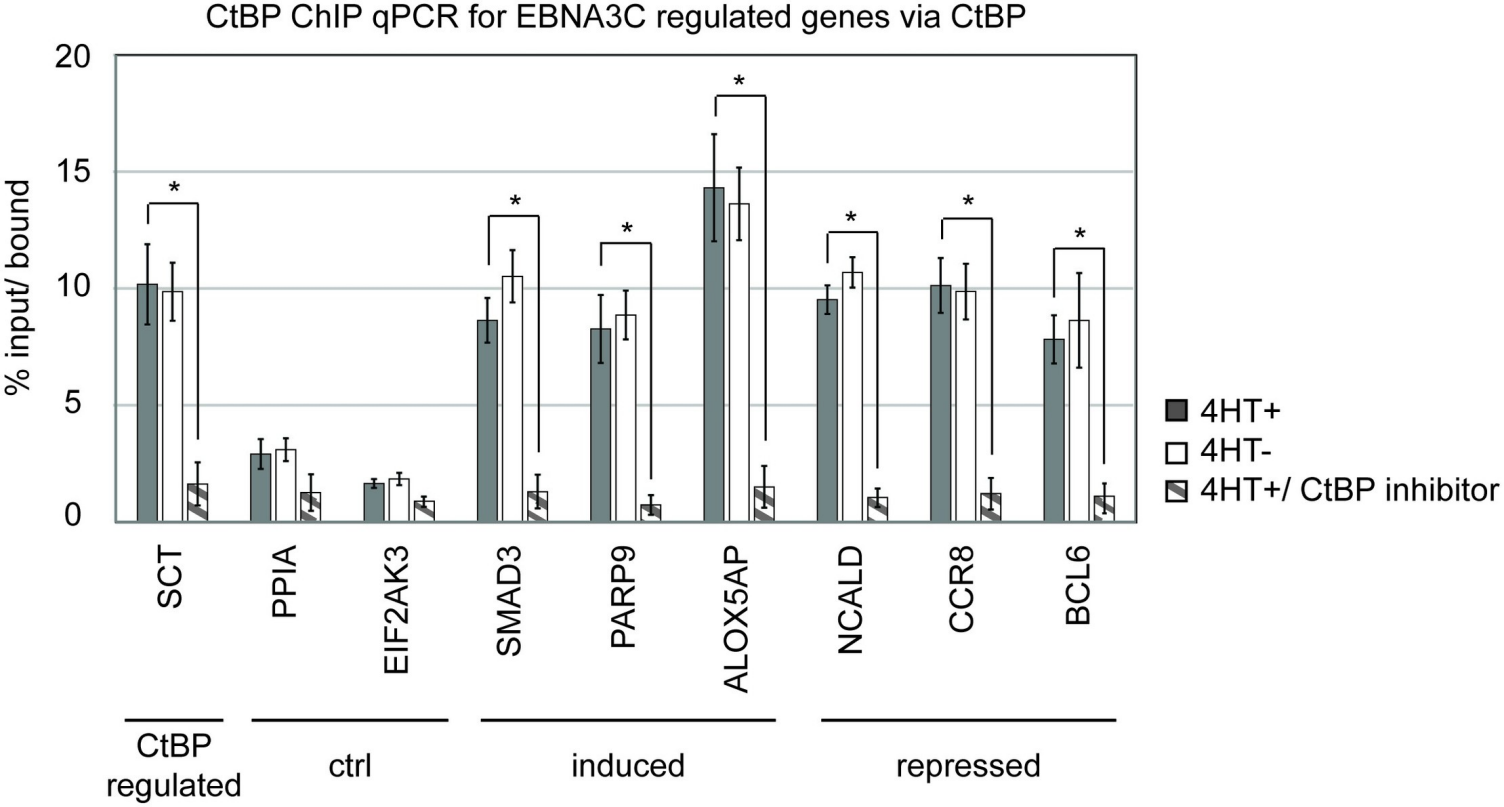
CtBP occupancy is not regulated by EBNA3C. ChIP qPCR was conducted to determine CtBP1 occupancy at the promoters of the indicated EBNA3C regulated genes. Results from CDKN2A-ex2-KO EBNA3C-HT LCLs grown in the presence (dark gray bar) or absence of 4HT (white bar) for 2 weeks or the presence of 4HT and the CtBP inhibitor HIPP (gray striped bar). Purified DNA was quantified using indicated gene specific primers (control genes, EBNA3C induced genes, or EBNA3C repressed genes) and plotted as percent bound relative to input. This experiment is representative of two independent biological replicates and stastically significant differences (p-value < 0.05 by two-sample t-test) are indicated (*).

The importance of the CtBP repressor for mediating EBNA3C upregulation of host genes was unexpected. Recruitment of CtBP to promoters by EBNA3C has been proposed as a mechanism of EBNA3C mediated repression, but it less clear how EBNA3C might interact with CtBP to activate gene expression. To further examine this phenomenon, we performed chromatin precipitation for CtBP in the presence and absence of functional EBNA3C. As an initial control, we examined CtBP binding at the SCT gene whose expression is known to be CtBP regulated [39]. We observed strong CtBP1 binding at SCT that was disrupted by the addition of the HIPP CtBP dimerization inhibitor [40,41] (Fig 6). CtBP1 binding was also detectable at EBNA3C induced genes, including the CtBP-dependent SMAD3 gene and the partially CtBP-dependent PARP9 and ALOX5AP genes (Fig 6). CtBP1 binding was also evident at the EBNA3C repressed genes NCALD, CCR8, and BCL6. We observed much weaker CtBP1 binding signals at the non-EBNA3C regulated control genes PPIA and EIF2AK3. In all cases, observed CtBP1 binding signals were disrupted by the CtBP dimerization inhibitor HIPP, confirming the specificity of our ChIP for CtBP1 binding at these sites. In contrast to the results obtained with the CtBP dimerization inhibitor, CtBP1 binding was not affected by the presence or absence of EBNA3C (Fig 6, compare 4HT+ to 4HT-), even at genes regulated by EBNA3C in a CtBP-dependent manner. These data suggest that, at least at these EBNA3C regulated genes, the CtBP repressor is not recruited by EBNA3C, but is pre-bound and EBNA3C’s CtBP binding domain may contribute to its ability to associate with regulatory elements.

### CtBP tonically represses host genes that are upregulated by EBNA3C in a CtBP-dependent manner

**Fig 7:**
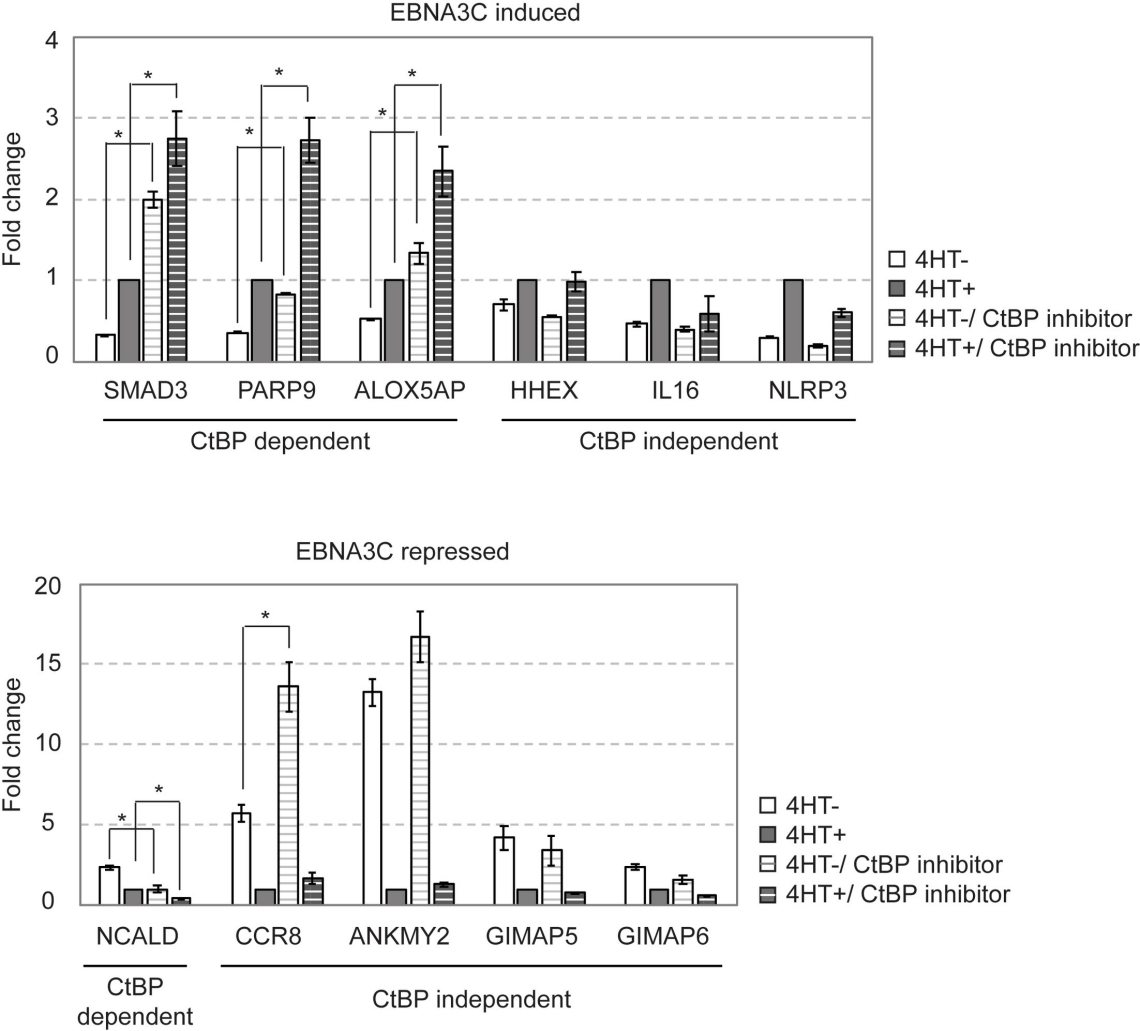
EBNA3C induced genes that require EBNA3C-CtBP interaction are also upregulated by chemical inhibition of CtBP. Relative expression of the indicated EBNA3C regulated genes was assessed by qRT-PCR in CDKN2A-ex2-KO EBNA3C-HT LCLs were cultured in the presence (4HT+, gray bar) or absence (4HT-, white bar) of EBNA3C activity for 2 weeks. Additionally, cells were treated with the CtBP inhibitor HIPP as indicated for 24 hours shown as striped bar. Expression level was normalized for each gene relative to the wildtype condition (i.e., 4HT+, no CtBP inhibition). Genes able to be regulated by EBNA3CmCtBP (CtBP independent) or not (CtBP dependent) are as indicated under the gene names. Statistically significant differences (p-values of <0.05 by two-sample t-test) are indicated (*). This experiment is representative of two biological replicates.

To further assess how CtBP mediates EBNA3C gene regulation, we measured mRNA levels of EBNA3C target genes in CDKN2A ex2-KO EBNA3C-HT LCLs in the presence or absence of 4HT from cultures treated with either the HIPP CtBP inhibitor or vehicle control. For each host gene, expression level was normalized to that observed in the presence of EBNA3C (Fig 7, 4HT+). In the case of EBNA3C induced genes (Fig 7, top panel), we observed clear upregulation by HIPP treatment for the subset of genes (SMAD3, PARP9, AlOX5AP) that were dependent or partially dependent on the EBNA3C-CtBP interaction. In contrast, HHEX, IL16, and NLRP3, which are induced equally well by either EBNA3C WT or EBNA3CmCtBP, were not upregulated by HIPP. These data imply that genes normally repressed by CtBP are susceptible to induction by EBNA3C.

For EBNA3C repressed genes, we did not see evidence of HIPP mediated de-repression for the partially CtBP-dependent NCALD gene (Fig 7, bottom panel). This was also true of the CtBP-independent GIMAP5 and GIMAP6 genes. Two other CtBP-independent EBNA3C repressed genes, CCR8 and ANKMY2 displayed varying degrees of HIPP de-repression (Fig 7, bottom panel, compare first and third bars); however, EBNA3C still exerted strong repressive effects even with CtBP inhibition (Fig 7, bottom panel, compare third and fourth bars). Taken together with our ChIP data, these results suggest that CtBP recruitment to EBNA3C target genes is not essential for gene repression, but that binding of EBNA3C near host genes repressed by CtBP can result in their upregulation.

### EBNA3C interaction with CtBP promotes p300 recruitment to EBNA3C induced genes

**Fig 8:**
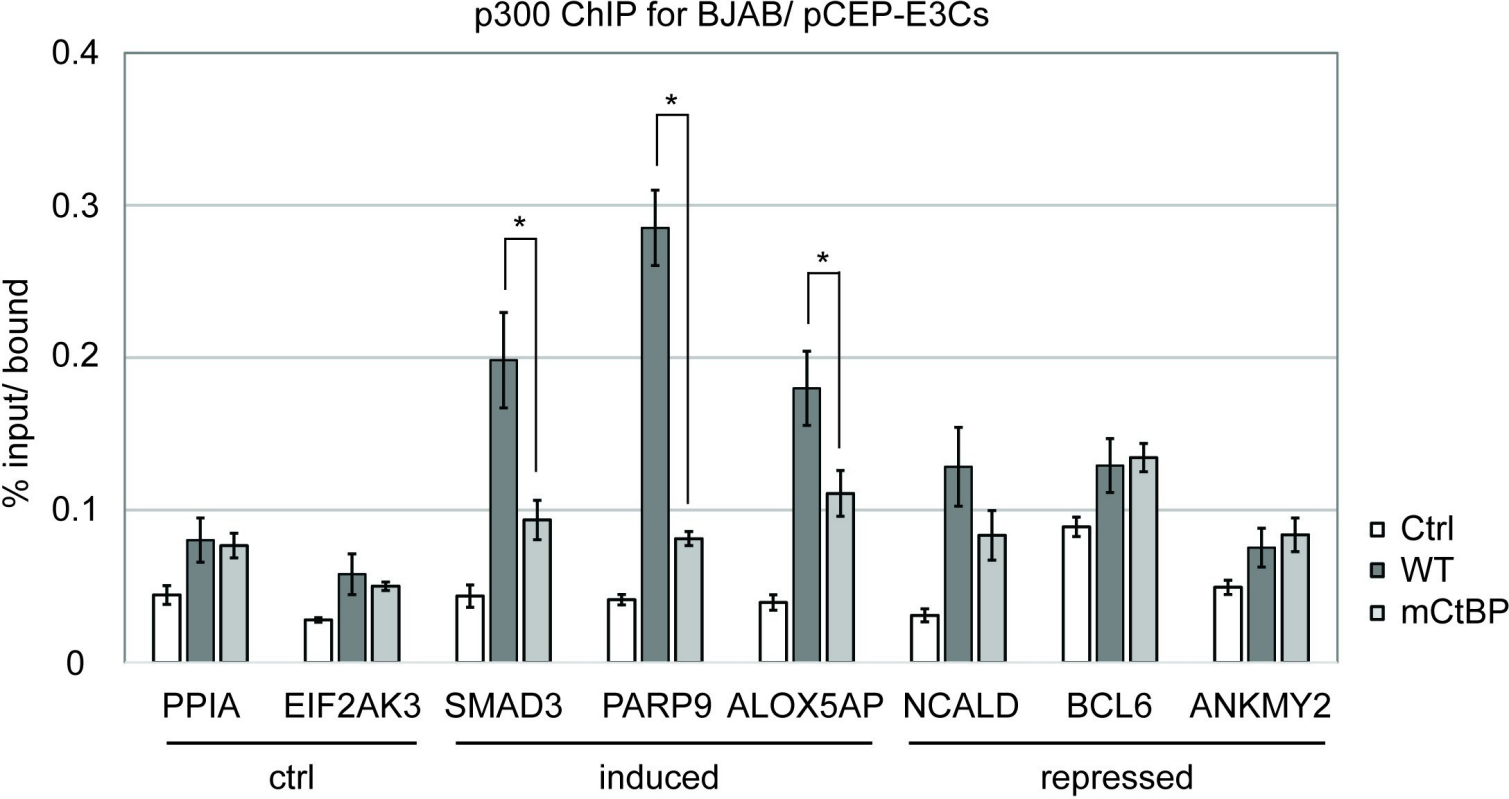
Loss of EBNA3C-CtBP binding decreases p300 occupancy at promoters of EBNA3C induced genes. Chromatin immunoprecipitation for p300 antibody was performed in BJAB/IRF4 cells stably expressing EBNA3C (WT), EBNA3C binding mutant (mCtBP) or vector control (Ctrl). Purified DNA was quantified using indicated gene specific primers (control genes, EBNA3C induced genes, or EBNA3C repressed genes) and plotted as percent bound relative to input. The experiment shown is representative of three biological replicates and statistically significant differences (p-value < 0.05) are indicated (*).

Because EBNA3C induced genes are characterized by increased histone acetylation, we hypothesized that EBNA3C might interfere with CtBP recruitment of histone deacetylases. To investigate this possibility, we performed ChIP for HDAC1 followed by qPCR for EBNA3C regulated genes in CDKN2A ex2-KO EBNA3C-HT LCLs in the presence or absence of 4HT. We did not detect any change in HDAC1 association with addition of 4HT at EBNA3C induced (SMAD3, PARP9, ALOX5AP), EBNA3C repressed (NCALD, CCR8, BCL6, ANKMY2, SETD7), or control genes (PPIA, EIF2AK3) (S5 and S6 Figs). Because CtBP is known to bind and repress the p300 acetyltransferase [42], we also investigated whether the EBNA3C-CtBP interaction could affect p300 recruitment. For these experiments, we utilized BJAB cell lines stably expressing IRF4 which we have previously demonstrated can model EBNA3C bound sites in LCLs [10]. We observed no difference in p300 association with the non-EBNA3C regulated control genes PPIA and EIF2AK3 (Fig 8) with stable EBNA3C co-expression. In contrast, at EBNA3C bound sites near the induced genes SMAD3, PARP9, and ALOX5AP, we observed ~2-fold increase in p300 association in the presence of EBNA3C, but minimal to no increase in the presence of EBNA3CmCtBP. This increased p300 association at EBNA3C bound sites was not observed at the repressed genes BCL6 and ANKMY2. Intriguingly, we did see evidence of EBNA3C dependent p300 recruitment to NCALD. Since we see clear evidence of EBNA3C repression of this gene in our earlier studies, it is likely that EBNA3C repressive mechanisms (e.g., H3K27me3 deposition) predominate at this gene over this up-regulatory effect. These data are consistent with the EBNA3C interaction with CtBP being important for p300 recruitment at a subset of EBNA3C induced genes.

## Discussion

In this manuscript we employ CRISPR/Cas9 editing to characterize the role of the EBNA3C transcription factor in LCL growth. Our results provide further evidence that repression of the p16 tumor suppressor gene is sufficient to compensate for loss of EBNA3C activity. We demonstrate, using two different CDKN2A exon knockouts, that EBNA3C regulated genes can be identified with much greater specificity when p16 activity is abrogated. Although several prior studies have reported EBNA3C regulated genes sets, there has been minimal concordance among them. Our results are particularly informative in that they allow a consensus EBNA3C regulome to be defined. Defining these genes is fundamental to discovering previously unknown roles of EBNA3C in B lymphocyte transformation (apart from its essential function of abrogating p16 induced growth arrest). We further determine specific genes that require RBPJ and/or CtBP interaction with EBNA3C for their regulation. Most significantly, our study provides surprising mechanistic insights into the role of the EBNA3C-CtBP interaction. Contrary to prior models, EBNA3C does not appear to promote CtBP recruitment, but instead interferes with CtBP repression activity to upregulate some host genes.

Previous studies have demonstrated that EBNA3C inactivation results in LCL growth arrest coincident with expression of the CDKN2A tumor suppressor gene products p16^INK4A^ and p14^ARF^ [21]. When first described, reversal of this growth arrest was found to require siRNA targeting both p16 and p14 or targeting of the Rb and p53 pathways by E7 and E6 respectively [21]. However, subsequent experiments using B lymphocytes from an individual with an inherited defect in p16 (p16-Leiden) found that they could be transformed by an EBNA3C null EBV [30]. While this suggests that p16 is the only essential target of EBNA3C for maintenance of LCL growth, there are two important caveats. First, the LCL transformation process may select against outgrowth of clones sensitive to p14 expression. Second, although the p16-Ledein fusion protein is argued to be intact for p14 function in the p53 pathway, it is disrupted for residues known to be important for non-canonical p14 activities that might affect LCL growth and/or survival [31–33]. These caveats and the discordant prior results prompted us to directly evaluate whether p16 disruption was sufficient to compensate for EBNA3C inactivation. Our findings firmly support the notion that p16 repression is sufficient to explain EBNA3C’s role in LCL growth maintenance. These results are further examples of an emerging trend in which gene inactivation by CRISPR/Cas9 has resolved discrepancies that arose from experiments reliant on RNA interference [43–45].

Unlike EBNA2 regulated genes, there has never been consensus regarding which host genes are subject to EBNA3C regulation. Although many factors likely contribute to this, the role of EBNA3 proteins as “fine-tuners” of the latency III program likely explains the sensitivity to various confounders of prior attempts at defining EBNA3C target genes. EBNA3 proteins compete with each other and EBNA2 for RBPJ binding and must therefore be studied at physiologically relevant expression levels [10,46,47]. Although LCLs are ideal for modelling these levels of expression, they undergo growth arrest upon EBNA3C inactivation [18]. Burkitt lymphoma (BL) cells, by contrast, do not depend on EBNA3C for continued growth [35]. However, we have previously shown that IRF4, a protein not expressed in germinal center B cells like BL, is important for EBNA3C regulation of many host genes [10]. Thus, it is not unexpected that genes found to regulated upon EBNA3C expression in EBV negative BL cells differ from those identified in LCLs. Despite the challenges imposed by EBNA3C inactivation in LCLs, two prior studies have used LCLs to identify EBNA3C regulated genes [30,34]. In the first, differentially expressed genes were identified in LCLs after 4HT withdrawal, but prior to growth arrest. The second employed LCLs derived from B cells with p16-Leiden which did not undergo growth arrest in the absence of EBNA3C activity. Remarkably, there was virtually no overlap in the genes identified in these two studies (see S3 Table). The CRISPR/Cas9 strategy employed in the current study convincingly demonstrates that p16^INK4a^ activity is the major driver of this discrepancy. First, we directly demonstrate (Fig 1D) that specificity in identifying EBNA3C target genes is only possible upon p16 inactivation. Second, all prior attempts to identify EBNA3C regulated genes resulted in distinct lists with minimal overlap. The EBNA3C regulated genes identified in our study are highly concordant with those reported by Skalska et al. Notably, the one feature in common between the two studies is the lack of p16 activity in the studied cell line–providing further evidence that this represents a major confounder to identification of direct EBNA3C targets. Indeed, the p16 effect appears to be more significant than methodological differences such as microarray vs. RNA-seq expression analysis. Despite the essential role of p16 regulation in EBNA3C effects, it is likely that other EBNA3C regulated genes play important roles in the virus lifecycle and EBV pathogenesis. A clear delineation of which genes are actually regulated by EBNA3C in LCLs is foundational to investigating these effects.

Our results are also important for demonstrating the extent to which EBNA3C alters B lymphocyte gene expression independent of its role in maintaining LCL growth. Our GSEA pathway analysis is consistent with substantial induction of interferon responsive genes by EBNA3C. This result is somewhat counterintuitive given prior reports of EBNA3C’s ability to downregulate multiple chemokines and our recent finding that EBNA3C decreases T-cell infiltration into EBV positive B cell lymphomas in a humanized mouse model [48,49]. In an effort to more clearly define the nature of apparent immune activation induced by EBNA3C, we conducted additional exploratory GSEA analysis using the C7 (Immunologic signatures) gene sets. This identified a strong correlation (NES 3.75, q-val 0) with a signature derived from comparison of naïve B lymphocytes with plasmablasts [50]. Remarkably, the core enriched genes from this signature include 20 of the 22 genes present in both the alpha and gamma interferon core enriched genes (S7 Fig). Thus, EBNA3C upregulation of genes induced by interferon signaling may in fact reflect a direct contribution of EBNA3C to EBV activation of naïve B lymphocytes into plasmablast-like LCLs and notably one that is independent of EBNA3C’s effect on growth. This also highlights the precision with which EBNA3C reprograms B lymphocytes as it also regulates genes such as p18^INK4c^ and PRDM1 to impede further differentiation into plasma cells [25]. Pathways exhibiting strong negative correlation with EBNA3C regulated genes included adipogenesis and hypoxia. In this case, distinct EBNA3C regulated genes contributed to the core enrichment in these pathways (Fig 4B). Additional gene sets of interest exhibiting slightly lower enrichment scores included: DNA_REPAIR (NES -1.9, q-val 0.03), UV_RESPONSE_UP (NES -2.1, q-val 0.011), OXIDATIVE_PHOSPHORYLATION (NES -2.2, q-val 0.008), MITOTIC SPINDLE (NES -2.2, q-val 0.008), E2F_TARGETs (NES -1.7, q-val 0.044), MYC_TARGETS_V2 (NES -1.7, q-val 0.046). The genes driving these signatures are potentially responsible for EBNA3C inhibition of the cell DNA-damage response that is mediated by replicative stress from early latency IIb mediated cell hyperproliferation [8,26,51,52].

In an effort to better define how EBNA3C regulates specific host genes, we trans-complemented our EBNA3C-HT CDKN2A ex2 KO LCLs with EBNA3C mutants defective for interaction with RBPJ or CtBP. Here again, the sensitivity of EBNA3C gene regulation to experimental conditions proved limiting, but we were nevertheless able to identify specific genes that were dependent or independent upon RBPJ interaction as well as upon the CtBP interaction. Our results are consistent with RBPJ being the more important interacting partner for EBNA3C effects on host gene expression. This strong dependence upon the RBPJ interaction belies the fact that only about 40% of EBNA3C bound sites colocalize with RBPJ [10,11]. This may suggest that RBPJ bound sites are functionally more significant that other EBNA3C bound sites. Alternatively, if multiple EBNA3C bound sites contribute to regulation of a gene, loss of the RBPJ co-bound subset may impair regulation of many genes despite continued binding of EBNA3C at sites not requiring the RBPJ interaction. The identification of specific genes that are regulated independent of RBPJ is also a significant advance. It will be important to determine if these are regulated via IRF4 [10]. If not, it would suggest an even greater array of host transcription factors mediate functionally important EBNA3C chromatin association. One unexpected finding was that COBLL1 was among our putative RBPJ-independent genes. Prior work has shown that COBLL1 was not able to be regulated by an EBNA3C mutant defective for RBPJ interaction [22]. Importantly, the mutant used in that study disrupted a second RBPJ interaction domain whose functional significance has not been established but is preserved in our EBNA3CmRBPJ mutant. The fact that our mutant retained the ability to regulate COBLL1, albeit at reduced efficiency, suggests that this second RBPJ interacting domain may have functional significance despite its inability to mediate p16 repression or interfere with EBNA2 activation of the EBNA (Cp) promoter [53]. Further studies assessing the ability of these two mutants to associate with chromatin and regulate cell genes may clarify this issue.

In contrast to RBPJ, loss of CtBP interaction had less of an impact on the gene regulation by EBNA3C (Fig 5C and 5D). Unexpectedly, interaction with the CtBP repressor proved important for EBNA3C induction of many host genes. Previous models have suggested that EBNA3C may recruit CtBP to promoters via its C-terminal PLDLS motif in order to down regulate target genes [17]. Although the EBNA3C-CtBP interaction is important for repression of CDKN2A, direct recruitment by EBNA3C has not been observed [23]. Our data are consistent with this interaction contributing to the repression of NCALD and possibly other host genes, but we could not find evidence that CtBP occupancy was increased by nuclear localization of EBNA3C. Instead, our results suggested that CtBP binds to target genes independent of EBNA3C and the PLDLS motif is important for EBNA3C’s ability to upregulate genes. Notably, these genes were also subjected to tonic repression by CtBP as evidenced by their upregulation by HIPP, a CtBP inhibitor [40,41]. Although the mechanism of this interference remains to be fully defined, our results suggest that EBNA3C reverses epigenetic repression via recruitment of the p300 coactivator to a subset of genes repressed by CtBP.

EBNA3C’s ability to interfere with CtBP functions may also be responsible for the negative enrichment of hypoxia induced genes observed in our GSEA analysis. Because hypoxia is associated with high NADH/NAD+ ratios, it is among the conditions that promote CtBP dimerization and repression [16]. Aerobic glycolysis is another state associated with high NADH/NAD+ ratios and EBNA3C interference with CtBP effects may be part of the mechanism by which it attenuates the replicative stress associated with latency IIb hyperproliferation [52]. Although genes subject to CtBP repression have been reported from studies of the MCF-7 breast cancer cell line [54], CtBP target genes in LCLs have not been reported. It will be interesting to determine the extent to which these are also regulated by EBNA3C. Finally, it is noteworthy that hypoxia is also a state that promotes EBV entry into lytic replication [55]. Thus, by at least two independent mechanisms (control of plasma cell differentiation and inhibition of the hypoxia response), EBNA3C acts to maintain a state of viral latency in addition to its role in promoting latency III associated B lymphocyte growth.

## Materials and methods

### Cell culture and reagents

CDKN2A-ex1α-KO and ex2-KO EBNA3C-HT LCLs were derived from EBNA3C-HT LCLs [18] by CRISPR/Cas9 gene editing (as detailed below) and cultured in RPMI1640 (Gibco) supplemented with L-glutamine (Gibco), penicillin-streptomycin (Gibco), 10% Fetal Bovine Serum (FBS, VWR International) with or without 200 nM 4-hydroxytamoxifen (4HT, Sigma). BJAB/IRF4 and BJAB/IRF4/EBNA3C cells [10] were cultured in RPMI 1640 which supplemented with L-glutamine, penicillin-streptomycin, and 10% FBS. When required, CtBP inhibition was achieved by addition of 4mM 2-hydroxyimino-3-phenylpropanoic acid (HIPP, Sigma) [40,41] to culture medium for 24 hours.

### Plasmids

pCEP-Blasticidin was created by replacing the hygromycin phosphotransferse gene from pCEP4 (Thermo Fisher Scientific) with the Blasticidin resistance gene from pcDNA6/His (Life tech, Addgene). pCEP-Blasticidin was digested with XhoI/PciI and re-ligated with the SalI/BsiWI fragment (EBNA3C N-terminus) and BsiWI/Pci fragment (EBNA3C C-terminus) from either pCEP-EBNA3C-WT, pCEP-EBNA3C-mRBPJ and pCEP-EBNA3C-mCtBP [19,20], to make the appropriate pCEP-Blasti-EBNA3C expression vectors. To construct the pCEP-Blasti-mCherry plasmid, mCherry gene (Takara Bio) was cloned into pCEP-Blasticidin. To obtain pCEP-EBNA3C mCtBP F-HA, pCEP-Blasti-EBNA3C mCtBP was digested with XhoI/BsrGI and cloned into the pCEP-EBNA3C F-HA plasmid [10].

### Antibodies

The following antibodies were used for immunoblotting; p16 (clone JC8, sc-56330, Santa Cruz Biotechnology), p14 ARF (Santa Cruz sc-8613), beta Actin (Santa Cruz, sc-69879), Flag (Sigma, F1804), AICDA (Cell signaling, #4975), SMAD3 (Invitrogen, 51–1500), Rb1 (cell signaling, #9309), SETD7 (Cell signaling, #2825), and alpha Tubulin (Sigma, T6074), and chromatin immunoprecipitation; CtBP1 (EpiGentek, #A2705), HDAC1 (Thermo Fisher PA1-860), and p300 (Bethyl A300-358A) and p53 (Sant Cruz, sc126).

### CRISPR/Cas9 plasmids and knockout of CDKN2A-ex1α and CDKN2A-ex2

CRISPR/Cas9 plasmid cloning was performed as previously described [56]. Briefly, Cas9 mediated editing of p16^INK4A^ was accomplished by cloning either of two targeting 20mers for the gRNA (CDKN2A-ex1α-KO: GAGCAGCATGGAGCCGGCGG and CDKN2A-ex2-KO: CGGGTCGGGTGAGAGTGGCG) [57,58] into the pX330 plasmid [59]. The Cas9 expression cassette and gRNA were excised from pX330 by PciI/NotI digestion and cloned into pCEP4 with a modified polylinker sequence, which allowed for hygromycin selection via the self-maintaining episomal plasmid.

Five million EBNA3C-HT LCLs were harvested during log-phase growth, resuspended in 100ul of buffer V with pCEP-CRISPR- CDKN2A-ex1α-KO or CDKN2A-ex2-KO plasmid, transfected using program X-001 of Amaxa Nucleofector, and cultured for 2 days in RPMI1640 complete medium. 20,000 cells were plated in 96 well culture plates using RPMI1640 complete medium with 100ug/ml hygromycin for 3 weeks. Hygromycin resistant cells were harvested and screened with DNA PCR using primer pairs for CDKN2A-ex1α (F: CCTTGCCTGGAAAGATACCG and R: TGGCTCCTCATTCCTCTTCC) or CDKN2A-ex2 (F: GGGCTCTACACAAGCTTCCT and R: TCAGGCCGTCCCACCGATTG). These primers were also used for Sanger sequencing of PCR products. Hygromycin was then removed from culture media and cells passaged to allow for loss of oriP-CRISPR plasmids from cells [60].

### Western blot analysis

Total-cell lysates were separated by sodium dodecyl sulfate-polyacrylamide gel electrophoresis (SDS PAGE), blotted onto nitrocellulose membrane, and probed with appropriate antibodies. After extensive washing, horseradish peroxidase conjugated secondary antibodies (Jackson Immuno Research) were applied. After incubation for 1–2 hours the membrane was washed again and developed with chemiluminescence reagent (GE Healthcare). Western blots were visualized and analyzed on Bio-Rad ChemiDoc Imager (Bio-Rad).

### Quantitative reverse transcription polymerase chain reaction (qRT-PCR)

The extracted RNA was treated with DNase, followed by reverse transcription using random primers and GoScript reverse transcriptase (RT) (Promega). Quantitative PCR was performed on the reverse-transcribed cDNA using iTaq Universal SYBR green mix (Bio-Rad) in a Bio-Rad CFX96 machine. 2ul of cDNA was used for 40 cycles (15 seconds at 95°C and 30 seconds at 60°C) using primers as shown in S5 Table. Fold change was calculated with the delta-delta Cq method; Cq numbers were normalized with TBP (delta-delta Cq) and divided with 4HT positive or negative (set as 1). The experiment shown is representative of two independent experiments and error bar indicating standard error of the mean within experiments.

### RNA-seq library prep and analysis

RNA samples were harvested from CDKN2A-ex1α-KO or CDKN2A-ex2-KO EBNA3C-HT LCLs in the presence or absence of 4-hydroxytamoxifen (4HT) for 2 weeks or pCEP-Blasti-EBNA3C trans-complementated CDKN2A-ex2-KO EBNA3C-HT LCLs. RNA-seq libraries were prepared by using Illumina TruSeq stranded total RNA kit (Illumina) and sequencing was performed by the University of Wisconsin-Biotechnology Center DNA Sequencing Facility. After assessing quality with FastQC (v.0.11.5), reads were mapped to GRCh38 using STAR 2.5.1b with the two-pass method described in the GDC Harmonized mRNA Analysis Pipeline. Read quantification was performed with featureCounts v.1.6.2 against the GENCODE v22 annotation. Differential gene expression analysis was performed in Galaxy with DESeq2 1.18.1. Gene set enrichment analysis was performed with GSEA PreRanked (ranking metric: -log(p-value)*sign(logFC) with MSigDB Hallmark gene sets v7.1. Confirmatory qRT-PCR and western blotting were performed for randomly selected genes. RNA-seq datasets described in this study have been deposited in the NIH SRA under BioProject accession PRJNA683081.

### Complementation assay

Five million of CDKN2A-ex2-KO EBNA3C-HT LCLs were cultured for 2 weeks in the absence of 4HT, harvested during log-phase growth, transfected with 2ug of pCEP-blasti-EBNA3C plasmid DNA using program X-001 of Amaxa Nucleofector (Lonza), and cultured for 2 days in RPMI1640. Cells were then washed with PBS and cultured in complete medium with 4ug/ml blasticidin for 2 weeks. RNA samples were isolated from blasticidin resistant populations and qRT-PCR or RNA-seq were conducted. RNA-seq libraries were prepared by using Illumina TruSeq stranded total RNA kit and analyzed as described above.

### Chromatin immunoprecipitation (ChIP) and quantitative PCR

ChIP assays were performed as described previously [56]. Briefly, 1 to 2x10^6^ cells (approximately 25ug sheared DNA) per ChIP were fixed in 1% (wt/vol) formaldehyde and sonicated using cup horns sonication system (Qsonica). After lysate clearing by centrifugation, supernatants were diluted and incubated with protein A/G magnetic beads with for 1 hour with rotation at 4°C. Protein A/G magnetic beads were removed and supernatants were used in ChIP experiments. Two to five micrograms of antibody or 20ul of HA magnetic beads were added per ChIP reaction, followed by incubation overnight at 4°C with rotation. Purified DNA was quantified using gene specific primers and iTaq universal SYBR green supermix (Bio-Rad) using a CFX96 touch real-time PCR detection system (Bio-Rad). Purified input DNAs were used in real-time PCR for standardization. Primers used for these experiments were as shown in S5 Table. The experiment shown is representative of two independent experiments and error bar indicating standard error of the mean within experiments.

## Supporting information

S1 Fig Confirmation of CRISPR/Cas9 gene editing of CDKN2A exon 2. (A) Candidate clones were screened by PCR using primers flanking the region of exon 2 targeted by the sgRNA (see materials and methods for oligonucleotide sequences). (B) Sanger sequencing for each of the two bands obtained for clone 3 (c3) revealed disruption of both alleles. (C) Western blot for Cas9-flag expression in EBNA3C-HT LCLs transfected with pCEP-CRISPR CDKN2A ex2 plasmid (+) or not (-). CDKN2A ex2-KO EBNA3C-HT LCLs were grown in the absence of hygromycin selection for at least two weeks.
(TIF)

S2 Fig Comparison of p14 fusion proteins created by Leiden mutation and CRISPR/Cas9 editing. (A) Multiple sequence alignment of p14^ARF^ and predicted amino acid sequences of p14 mutant proteins created by the p16 Leiden mutation or CRISPR/Cas9 editing in this study. Sequences implicated in canonical p14^ARF^ activities are intact in all fusion proteins. Residues downstream of exon 1α implicated in non-canonical p14^ARF^ functions are disrupted as shown in red. (B) Western blot for p53 and beta actin of lysates from EBNA3C-HT and EBNA3C-HT ex2-KO LCLs grown in the presence or presence of 4HT for 2 weeks.
(TIF)

S3 Fig Confirmation of CRISPR/Cas9 gene editing of CDKN2A exon 1α. (A) Candidate clones were screened by PCR using primers flanking the region of exon 1α targeted by the sgRNA (see materials and methods for primer sequences) and exon 2 as a control. (B) Sanger sequencing of the resultant product for the clone used in this manuscript revealed a 24bp deletion that included the initiation ATG codon. It is unknown if the absence of a second product is due to a much larger deletion of the second allele or loss of heterozygosity.
(TIF)

S4 Fig Confirmatory qRT-PCR to evaluate dependence upon CtBP interaction for EBNA3C regulation of specific host genes. qRT-PCR results from ex2-KO EBNA3C-HT LCLs trans-complemented with EBNA3C (WT), EBNA3C CtBP binding mutant (mCtBP). Expression level (relative to mCherry vector control) for the indicated EBNA3C induced or repressed genes is shown. Dependence upon CtBP interaction, as determined in the RNA-seq experiments, is indicated below the gene names.
(TIF)

S5 Fig HDAC1 occupancy on the promoter for EBNA3C regulate genes does not change upon EBNA3C inactivation. Chromatin Immunoprecipitation (ChIP) with HDAC1 antibody was conducted to determine whether EBNA3C disrupts CtBP/ HDAC1 repressor complex to enhance target gene expression. ex2-KO EBNA3C-HT LCLs cells were cultured in the presence or absence of 4HT for 2 weeks and ChIP with HDAC1 antibody followed qPCR for promoter sequences of the indicated genes was performed.
(TIF)

S6 Fig Genomic context of EBNA3C regulated genes. EBNA3C and RBPJ binding signals in EBNA3C regulated genes from previously published ChIP-seq experiments [10) displayed on the UCSC genome browser. Approximate locations of PCR primers used in this study are indicated with an asterisk (ChIP qPCR primer) or a cross (qRT-PCR primer).
(TIF)

S7 Fig Comparison of GSEA IFN signatures with a high scoring immunologic gene set signature. Right panel: Running enrichment plot for EBNA3C regulated genes versus the C7 gene set NAIVE_BCELL_VS_PLASMABLAST_UP. Left panel: Venn showing the extent of overlap of core enriched genes among the indicated gene sets. Note that the naïve B cell versus Plasmablast core enriched genes account for 20 of 22 genes in common between the interferon gamma and alpha signatures.
(TIF)

S1 Table Differential expression analysis of host genes upon EBNA3C inactivation in CDKN2A-ex2-KO EBNA3C-HT LCLs. Columns correspond to Entrez gene symbol (Symbol), its mean expression level (BaseMean), log2 fold increase in presence of EBNA3C [HT+/HT-] (log2 Fold Change), log fold change standard error (lfcSE), Wald statistic, raw p-value, and p-value adjusted for multiple testing. Table is sorted by increasing adjusted p-value and those less than 0.05 are included in Fig 3A.
(XLSX)

S2 Table Differential expression analysis of host genes upon EBNA3C inactivation in CDKN2A-ex1α-KO EBNA3C-HT LCLs. Columns correspond to Entrez gene symbol (Symbol), its mean expression level (BaseMean), log2 fold increase in presence of EBNA3C [HT+/HT-] (log2 Fold Change), log fold change standard error (lfcSE), Wald statistic, raw p-value, and p-value adjusted for multiple testing. Table is sorted by increasing adjusted p-value and those less than 0.05 are included in Fig 3A.
(XLSX)

S3 Table Comparison of EBNA3C regulated genes with previous studies. Table includes the high confidence 134 EBNA3C induced and 87 EBNA3C repressed genes corresponding to the intersections of the Venn diagrams in Fig 3A. For each gene the table reports: Entrez gene number (Number), Entrez Symbol (Symbol), log2 fold changes in response to EBNA3C and adjusted p-values from our study, and log2 fold changes in response to EBNA3C from the studies of Zhao, et al. 2011 and Skalska, et al. 2013 as indicated.
(XLSX)

S4 Table Summary of dependencies upon RBPJ or CtBP interaction for regulation by EBNA3C. Left table lists EBNA3C induced genes according to whether their regulation required the EBNA3C-RBPJ interaction or the EBNA3C-CtBP interaction. Genes listed as DEPENDENT could not be regulated by EBNA3CmRBPJ (second column) or EBNA3CmCtBP (third column). For genes listed as INDEPENDENT there was no impaired regulation with EBNA3CmRBPJ (second column) or EBNA3CmCtBP (third column). Genes listed as PARTIAL exhibited partially impaired regulation with the appropriate mutant. Note that the majority of the 134 EBNA3C induced and 87 EBNA3C repressed genes were not able to be classified in this analysis.
(XLSX)

S5 Table Sequences of PCR primers used in this study. **Primer pairs used for qRTPCR and ChIP qPCR are reported with the Entrez gene symbol of the transcript or regulatory region of the gene they were used to investigate.** For each pair, the primer sequence is reported or, in the case of previously published primers, the appropriate reference.
(XLSX)
