# Supplementary figures and images for: Epstein-Barr virus nuclear antigen 3C (EBNA3C) interacts with the metabolism sensing C-terminal binding protein (CtBP) repressor to upregulate host genes
Source: PLoS Pathog. 2021 Mar 15;17(3):e1009419. doi: 10.1371/journal.ppat.1009419 (PMC7993866; doi:10.1371/journal.ppat.1009419)

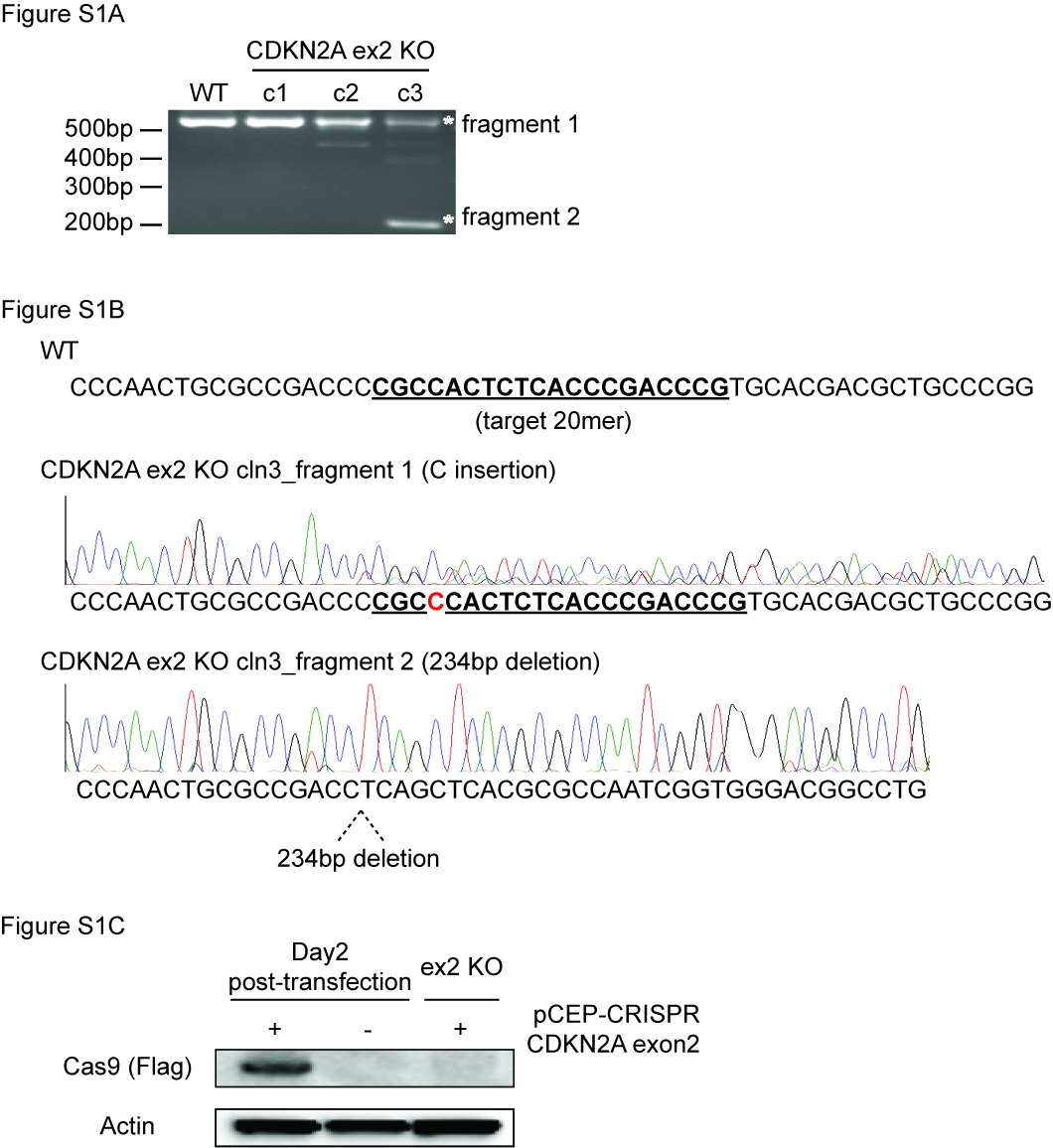

Supplement: S1 Fig — (A) Candidate clones were screened by PCR using primers flanking the region of exon 2 targeted by the sgRNA (see materials and methods for oligonucleotide sequences). (B) Sanger sequencing for each of the two bands obtained for clone 3 (c3) revealed disruption of both alleles. (C) Western blot for Cas9-flag expression in EBNA3C-HT LCLs transfected with pCEP-CRISPR CDKN2A ex2 plasmid (+) or not (-). CDKN2A ex2-KO EBNA3C-HT LCLs were grown in the absence of hygromycin selection for at least two weeks. (TIF) [file ppat.1009419.s001.tif]

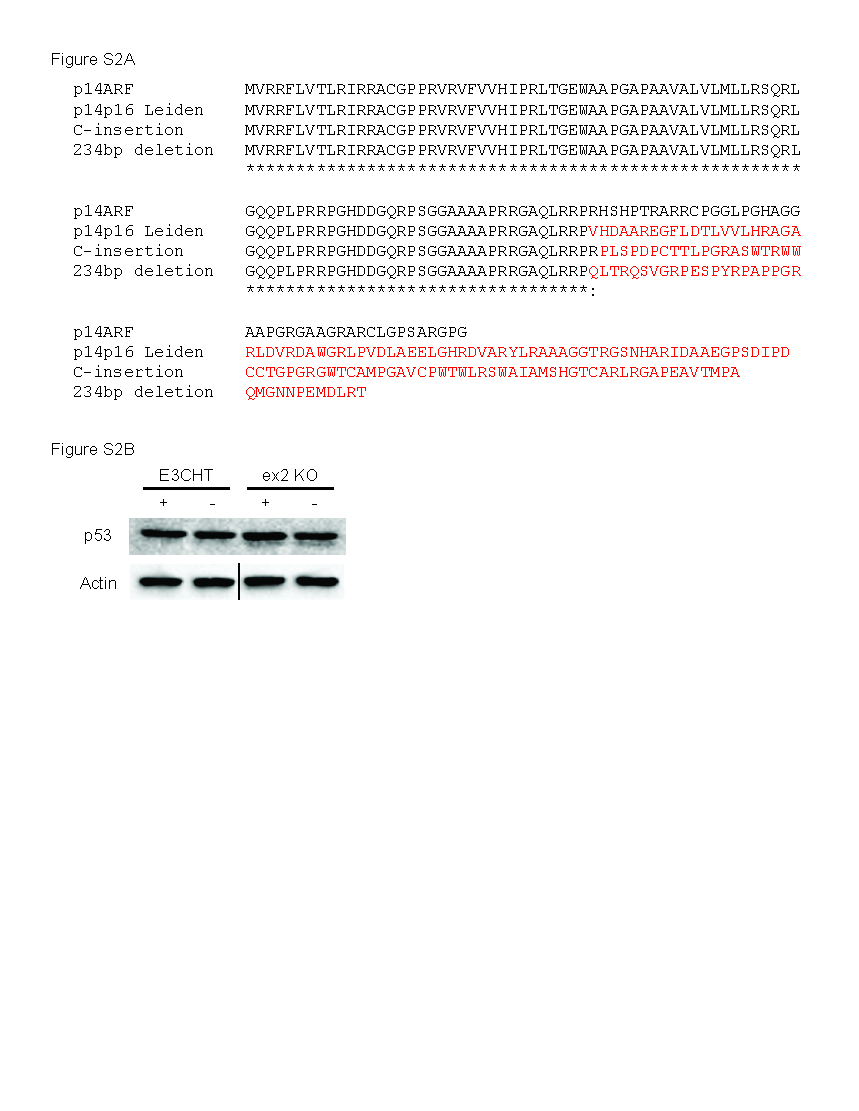

Supplement: S2 Fig — (A) Multiple sequence alignment of p14ARF and predicted amino acid sequences of p14 mutant proteins created by the p16 Leiden mutation or CRISPR/Cas9 editing in this study. Sequences implicated in canonical p14ARF activities are intact in all fusion proteins. Residues downstream of exon 1α implicated in non-canonical p14ARF functions are disrupted as shown in red. (B) Western blot for p53 and beta actin of lysates from EBNA3C-HT and EBNA3C-HT ex2-KO LCLs grown in the presence or presence of 4HT for 2 weeks. (TIF) [file ppat.1009419.s002.tif]

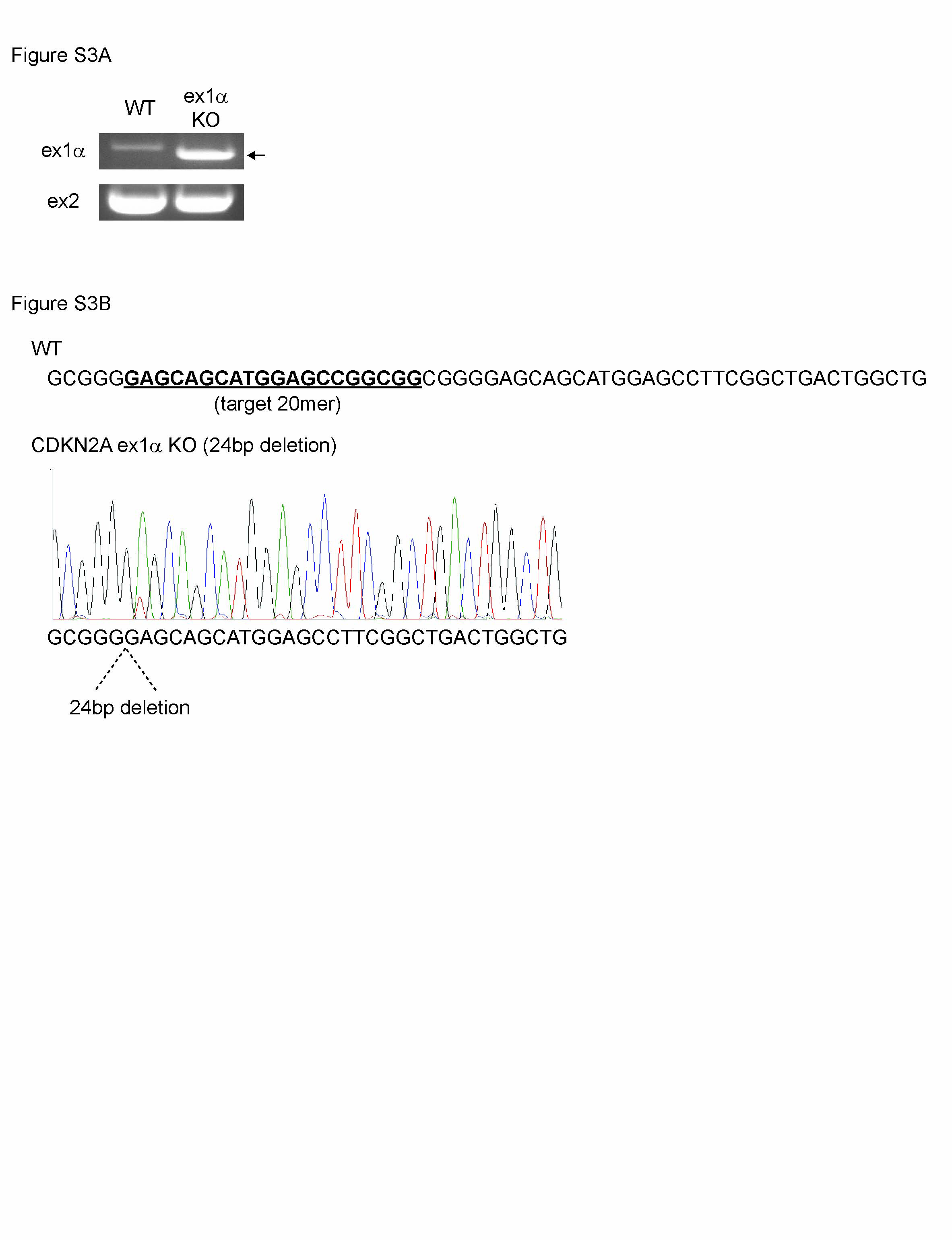

Supplement: S3 Fig — (A) Candidate clones were screened by PCR using primers flanking the region of exon 1α targeted by the sgRNA (see materials and methods for primer sequences) and exon 2 as a control. (B) Sanger sequencing of the resultant product for the clone used in this manuscript revealed a 24bp deletion that included the initiation ATG codon. It is unknown if the absence of a second product is due to a much larger deletion of the second allele or loss of heterozygosity. (TIF) [file ppat.1009419.s003.tif]

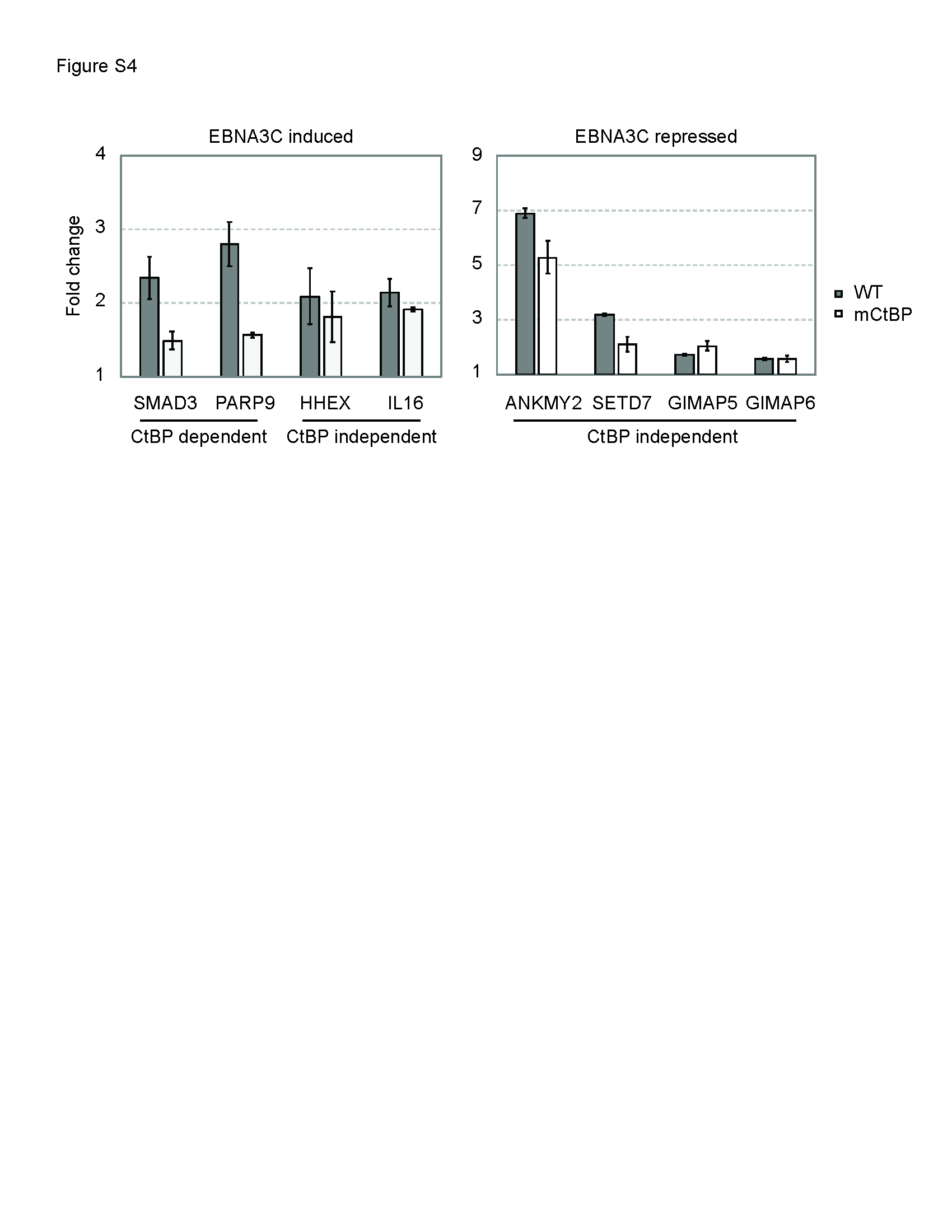

Supplement: S4 Fig — qRT-PCR results from ex2-KO EBNA3C-HT LCLs trans-complemented with EBNA3C (WT), EBNA3C CtBP binding mutant (mCtBP). Expression level (relative to mCherry vector control) for the indicated EBNA3C induced or repressed genes is shown. Dependence upon CtBP interaction, as determined in the RNA-seq experiments, is indicated below the gene names. (TIF) [file ppat.1009419.s004.tif]

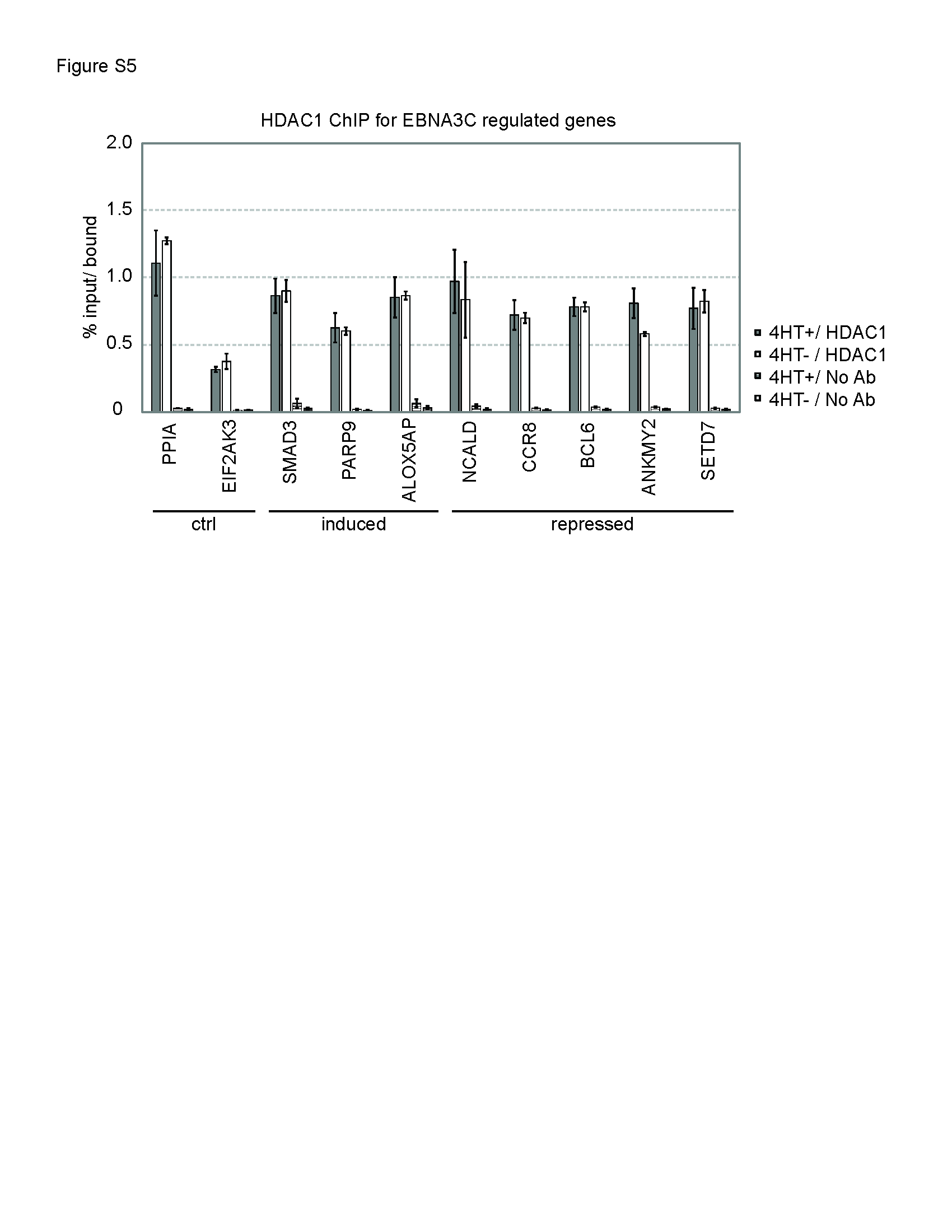

Supplement: S5 Fig — Chromatin Immunoprecipitation (ChIP) with HDAC1 antibody was conducted to determine whether EBNA3C disrupts CtBP/ HDAC1 repressor complex to enhance target gene expression. ex2-KO EBNA3C-HT LCLs cells were cultured in the presence or absence of 4HT for 2 weeks and ChIP with HDAC1 antibody followed qPCR for promoter sequences of the indicated genes was performed. (TIF) [file ppat.1009419.s005.tif]

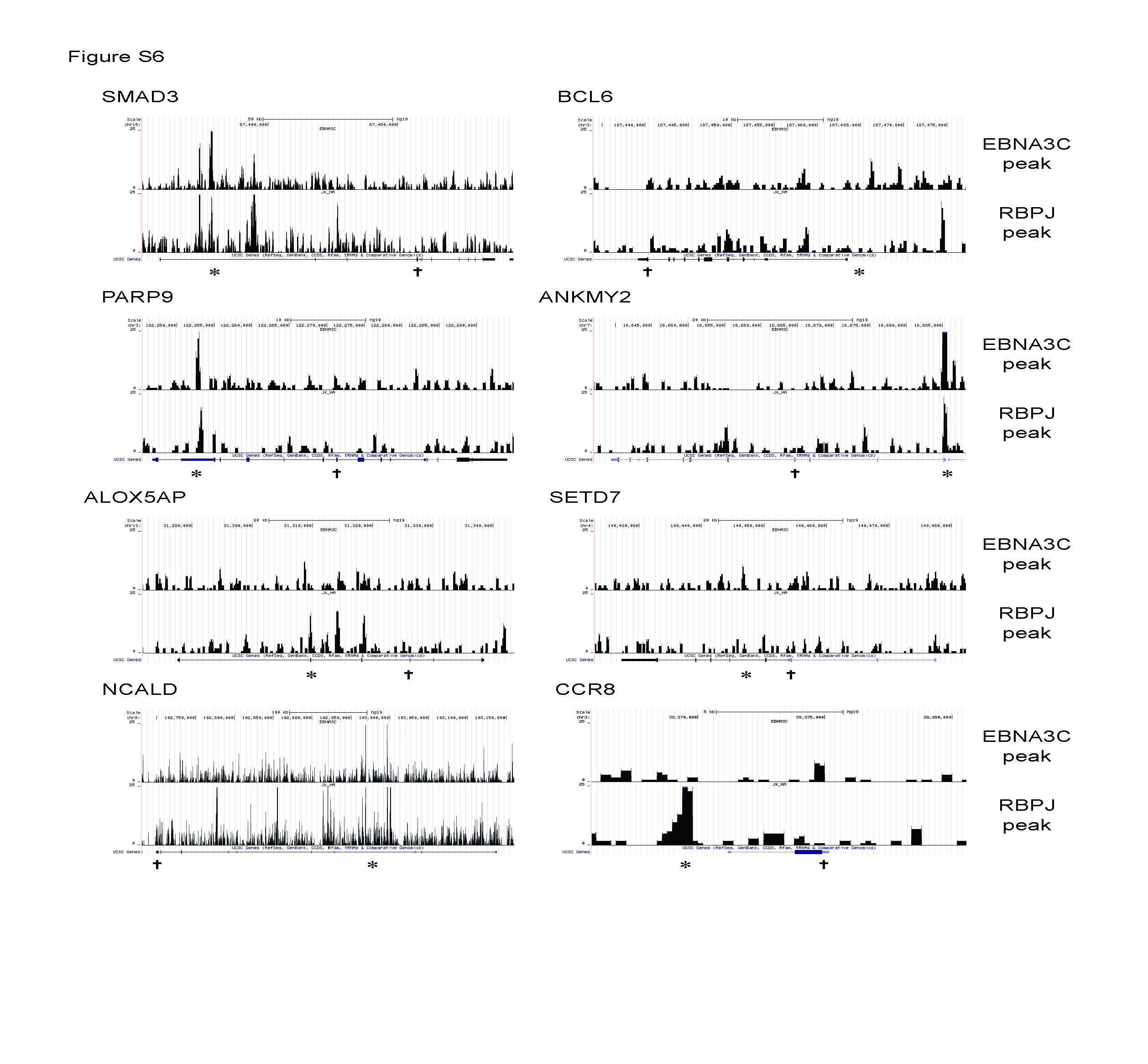

Supplement: S6 Fig — EBNA3C and RBPJ binding signals in EBNA3C regulated genes from previously published ChIP-seq experiments [10) displayed on the UCSC genome browser. Approximate locations of PCR primers used in this study are indicated with an asterisk (ChIP qPCR primer) or a cross (qRT-PCR primer). (TIF) [file ppat.1009419.s006.tif]

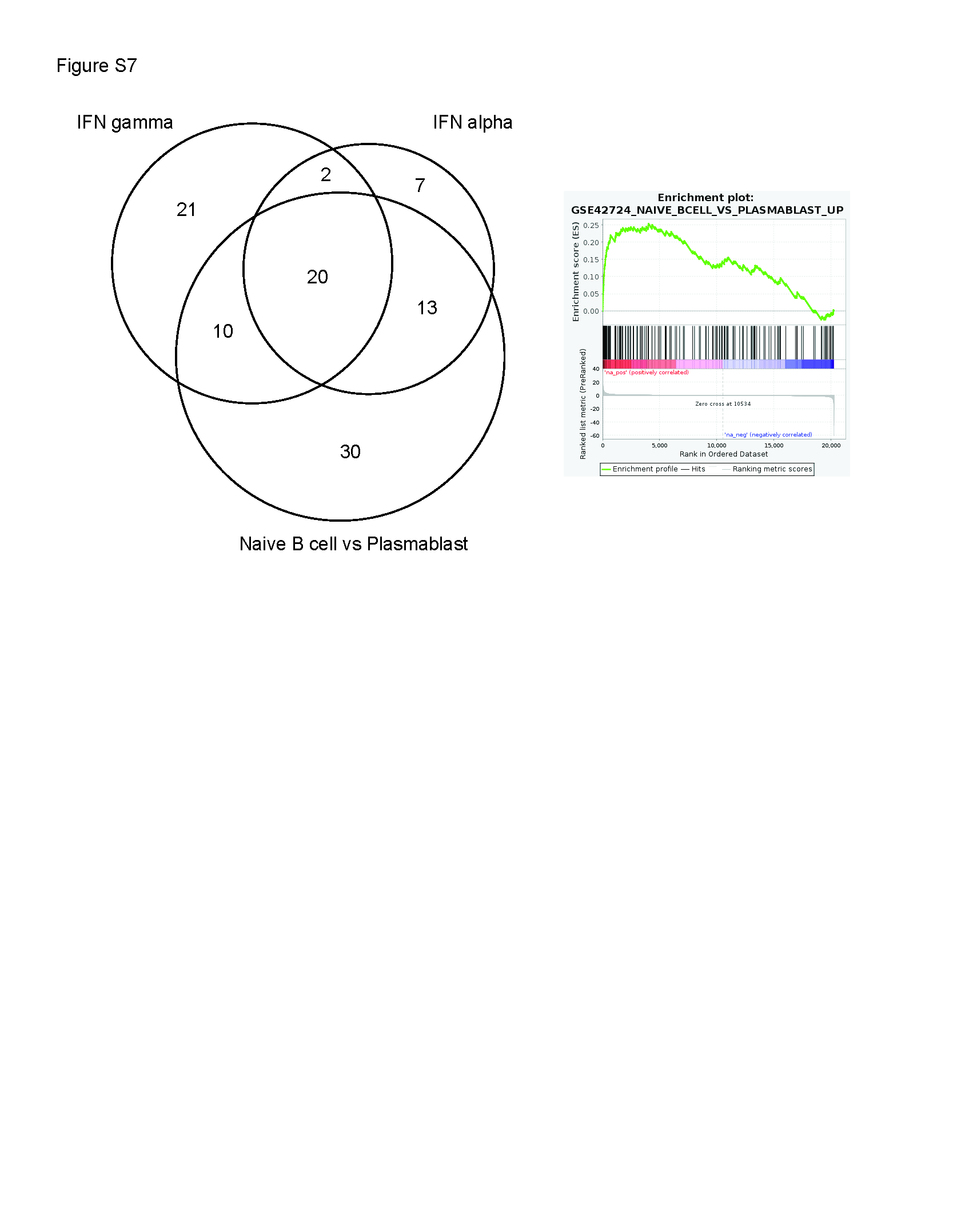

Supplement: S7 Fig — Right panel: Running enrichment plot for EBNA3C regulated genes versus the C7 gene set NAIVE_BCELL_VS_PLASMABLAST_UP. Left panel: Venn showing the extent of overlap of core enriched genes among the indicated gene sets. Note that the naïve B cell versus Plasmablast core enriched genes account for 20 of 22 genes in common between the interferon gamma and alpha signatures. (TIF) [file ppat.1009419.s007.tif]
